# A new approach for evaluating continuous and discontinuous pipeline deformation induced by soil tunnel excavation

**DOI:** 10.1038/s41598-023-38291-7

**Published:** 2023-08-04

**Authors:** Tao Yang, Bo Deng, Minghui Yang, Daxi Fu

**Affiliations:** 1 College of Civil Engineering, Hunan University, Changsha, 410082 Hunan Province People’s Republic of China; 2 College of Civil Engineering, University of South China, Hengyang, 421001 Hunan Province People’s Republic of China; 3 Department of Civil Engineering, Xiamen University, Xiamen, 361005 Fujian Province People’s Republic of China; 4 School of Civil Engineering, Beijing Jiaotong University, Beijing, 100044 People’s Republic of China

**Keywords:** Civil engineering, Engineering

## Abstract

The deformation of the overlying pipeline caused by the soil tunnel excavation cannot be ignored in the case of the small spacing between the pipeline and the tunnel. Based on the rigid bar method, the pipeline-soil interaction model was established, with the simply supported beam as the basic system, and the loads acting on the pipeline by the soil are considered to be linearly distributed. Calculation methods for continuous and discontinuous pipeline deformations were established. The results calculated by the proposed method agree well with the experimental data of centrifuge tests and field data. Parametric study on the effect of the volume loss (*η* = 1%, 2%,3 %), rotational stiffness (*β*_0_ = 4.47 × 10^6^N⋅m/rad, 4.47 × 10^8^N⋅m/rad, 4.47 × 10^10^N⋅m/rad), ratio of pipeline section length to inflection point of soil settlement curve (*L*/*i*_s_ = 0.5, 1.0, 1.5, 2.0) and soil elastic modulus (*E* = 10 MPa, 30 MPa, 50 MPa) on the deflection and joint rotation angle of the discontinuous pipeline were carried out. Results show that: (1) the maximum pipeline deflection and the maximum rotation angle of the joint increase as *η* increases and decrease as *β*_0_ increases; (2) in the "odd" case, the maximum pipeline deflection and the maximum rotation angle of the joint first increase and then decrease as *L*/*i*_s_ increases, reaching a peak at *L*/*i*_s_ = 1.5, while in the "even" case, the maximum pipeline deflection decreases as *L*/*i*_s_ increases and the maximum rotation angle of the joint first increases and then decreases as *L*/*i*_s_ increases; (3) in the "odd" case, the maximum pipeline deflection and the maximum angle of rotation of the joint decrease as *E* increases, while the opposite trend is observed in the "even" case. Additionally, the maximum pipeline deflection and the maximum rotation angle of the joint are always greater in the "odd" case than that in the "even" case.

## Introduction

**Figure 1:**
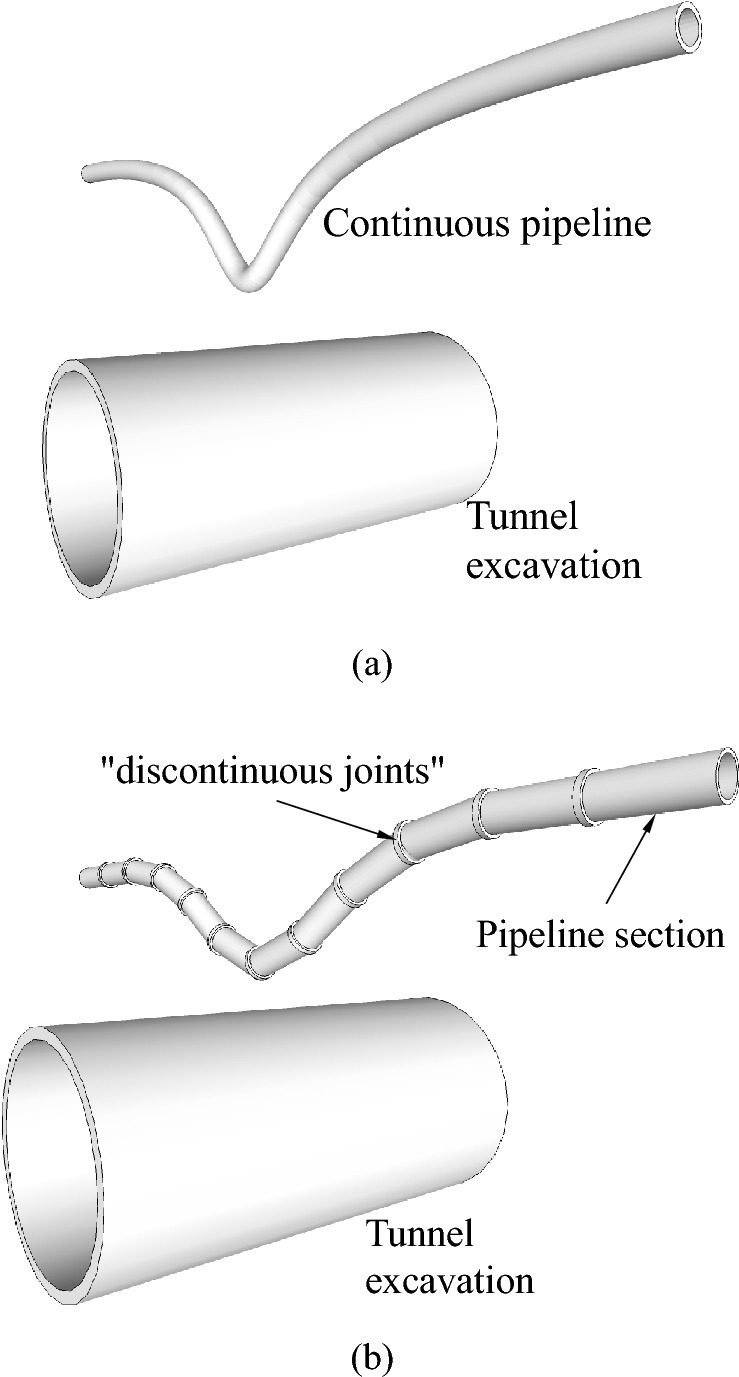
Case for tunnel excavation on existing pipelines: (**a**) continuous pipeline; (**b**) discontinuous pipeline.

The excavation of urban metro tunnels causes deformation of the surrounding soil, which in turn causes damage, leakage and interface detachment of pipelines in the soil layer, and even causes stratum cavity or ground collapse in serious cases, threatening the safety and stability of the city and the safety of people’s lives and property. For example, on 5 February, 2007, a construction section of Line 2 of the Nanjing Metro in Jiangsu Province, China, caused a buried gas pipeline to rupture and explode, resulting in the loss of water, electricity and gas to more than 5000 residents in the vicinity due to a lack of prior investigation of the surrounding gas pipeline and the lack of standard excavation work. Additionally, on 24 December, 2014, at Zongguan Station in Wuhan, Hubei Province, China, the excavation of the shield tunnel caused a local burst of an already aged water main line, resulting in water surges in the foundation pit and right line tunnel. Thus, the reasonable calculation of the deformation value of the overlying pipeline during the excavation of the soil tunnel has become one of the most concerned issues in this type of engineering, as shown in Fig. 1.

For the calculation of overlying pipeline deformation caused by tunnel excavation, the common methods to predict pipeline deformation include theoretical analysis^1–15^, numerical simulation^16,17^ and model test^18,19^. Compared with the other two methods, the theoretical analysis has significant advantages in practical application by virtue of its simplicity and convenience, so many scholars have carried out a lot of research on it and achieved rich results. For example, in 1986, Attewell et al.^1^ first used the Winkler foundation model to explore the impact of tunnel underpasses on existing underground pipelines. Wang et al.^2^ established a theoretical and analytical model of pipeline-soil interaction, obtained the analytical solution of pipeline deformation, and explored the law of pipeline-soil interaction. Klar et al.^3^ obtained an analytical solution for the Winkler elastic foundation beam for pipeline deformation due to tunnel excavation and compared it with the elastic continuum foundation solution, correcting the foundation coefficients for the Winkler elastic foundation beam. Vorster et al.^4^ gave a continuous elastic solution and verified its feasibility with centrifugal model tests. Shi et al.^5^ gave a solution for continuous pipeline deformation based on a two-parameter Pasternak foundation model using the energy variational method. Yang et al.^6^ solved for the pipeline deformation using the energy variation method on the assumption that both greenfield displacements and pipeline settlements conform to a Gaussian distribution. Fu et al.^7^ considered the phenomenon of pipeline-soil separation and used a two-parameter Parsternak foundation model to give a solution for pipeline deformation caused by tunnel excavation. The above studies on the effects of tunnel excavation on the overlying pipeline have mostly been carried out on the assumption that the pipeline is homogeneous and continuous, e.g. welded joint pipelines, cannot take into account the permissible rotation of the pipeline joints.

However, the assumption of continuity of the pipeline is now being questioned and the corresponding approach is being proposed to take into account the effect of rotation of the pipeline joints. Such as, Klar et al.^9^ abstracted the pipeline joints as joint elements capable of withstanding both bending moments and shear forces, while the pipeline sections between adjacent joints as beam elements, from which the stiffness matrices of the two elements are derived, and the boundary integral method is used to solve the deformation of the existing pipeline under the influence of tunnel excavation. Zhang et al.^10^ introduced "virtual nodes" at pipeline joints based on the Winkler foundation model and used the finite difference method to solve for the deformation of pipelines with joints under the influence of tunnel excavation. Dong et al.^12^ simplified the discontinuous pipeline to a continuous pipeline with local additional loads by means of the pulse function in the mathematical model, and used the finite difference method to solve for the pipeline deformation. Although the above methods can take into account the rotation of the joints, the solution process is complex and difficult to promote in practical engineering applications.

The previous research work demonstrates that discontinuous pipelines have not been sufficiently studied to obtain a simpler and more general approach. To this end, a new method is proposed based on the rigid bar method, which divides the pipeline into a series of simply supported beams, which can be used to predict tunnel excavation-induced deformation of continuous pipelines (joint stiffness in pipeline with the same rigidity as the pipeline, with continuous rotation angles on both sides of the joint, e.g. flanged welded pipelines) and discontinuous pipelines (weakened joint stiffness, with discontinuous rotation angles on both sides of the joint, e.g. socketed cement pipelines). The comparison with centrifuge experiments and field data verifies the correctness of the method in this paper, which has certain guiding significance for practical engineering.

## Computational models and assumptions

The rigid bar method was proposed by Zemochkin and is mainly used for solving elastic foundation beam problems^20,21^. The method replaces the continuity link between beam and foundation soil with a finite number of rigid bars, and it is still one of the frequently used methods for engineering design because it is applicable to various foundation conditions and complex stresses of variable-section beams. In this paper, the rigid bar method is used as the basis for solving the deflection of overlying continuous and discontinuous pipelines induced by soil tunnel excavation.

**Figure 2:**
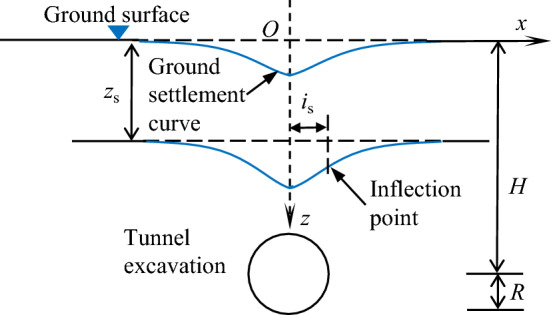
Soil settlement caused by tunnel excavation.

**Figure 3:**
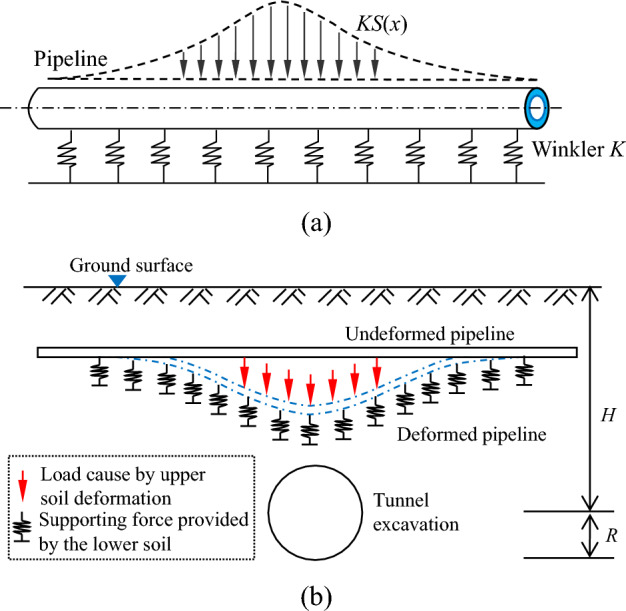
Calculation model for pipeline-soil interaction: (**a**) additional loads acting on the pipeline; (**b**) continuous pipeline deformation.

Due to the complexity of the tunnel-pipeline-soil interaction in the process of tunnel excavation, it is difficult to use direct modeling for analysis, and most of them use the two-stage method for approximate theoretical analysis. The first stage is to calculate the vertical displacement of soil caused by tunnel excavation at the pipeline axis (ignoring the influence of pipeline), as shown in Fig. 2. On this basis, the pipeline-soil interaction model is established, the soil deformation results are regarded as additional loads applied on the pipeline, and the Winkler foundation model has been used to analyze this type of problem, as shown in Fig. 3. In the second stage, the pipeline is regarded as a foundation beam, and the rigid bar method is used to divide the pipeline using the simply supported beam as the basic system. The support of the soil on the pipeline is regarded as a spring support concentrated at the joints, and the knowledge of structural mechanics is used to solve for the support reaction force and bending moment of each joint, which in turn completes the solution of the pipeline deflection and the rotation angle of the joint. The following assumptions exist in the solution process of this paper:

1. Pipelines outside the tunnel excavation impact area will not be affected.
2. The pipeline and soil are always in contact, and the volume loss remains unchanged.
3. The tunnel is not affected by the presence of the pipeline.
4. When the length of the pipeline is divided into smaller lengths, the additional load of the soil on the pipeline unit is considered to be linearly distributed.

### Tunnelling-induced greenfield settlements

According to the above ideas, the deformation of soil layer during tunnel excavation can be calculated first. Peck^22^ collected and compiled data from a large number of engineering examples and found that the greenfield settlement curve perpendicular to the tunnel axis due to volume loss during tunnel excavation roughly conforms to the following function (see Fig. 2):

1 $$S\left( x \right) = S_{\max } \exp \left[ { - x^{2} /\left( {2i_{{\text{s}}}^{2} } \right)} \right]$$

2 $$S_{{{\text{max}}}} = \pi R^{2} \eta /\left( {i_{{\text{s}}} \sqrt {2\pi } } \right)$$

3 $$i_{{\text{s}}} = 1.15R\left[ {H/\left( {2R} \right)} \right]^{0.9} \left( {1 - z_{{\text{s}}} /H} \right)^{0.3} $$

where *H* is the tunnel depth; *R* is the tunnel radius; *z*_s_ is the distance from soil layer to surface; *S*(*x*) is the soil settlement at *x*, *S*_max_ is the maximum value of soil settlement; *x* is the horizontal distance to tunnel axis; *η* is volume loss, *η* can also be determined by reference to existing similar tunnelling projects if it is difficult to obtain; *i*_s_ is the distance between the inflection point of the soil settlement curve and the centre of symmetry of the settlement curve, using the formula proposed by Jiang et al.^23^ as shown in Eq. (3).

### Computational models and theoretical background

Discontinuous pipelines in everyday life are usually flanged or socketed connections between pipeline sections. For the purpose of this analysis, the section-to-section joints are named "discontinuous joints".

**Figure 4:**
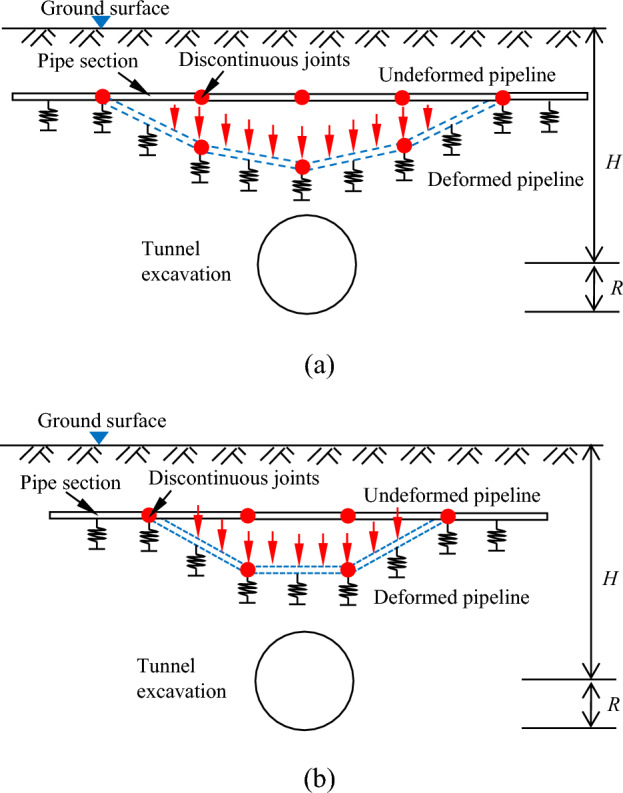
"Discontinuous joints" at different locations from the tunnel axis for (**a**) odd; (**b**) even.

As the position of the pipeline "discontinuous joints" changes with respect to the tunnel axis, the corresponding deformation of the pipeline will be changed. As such, Klar et al.^9^ considered two special cases and defined two positions of the "discontinuous joints" in relation to the tunnel axis: ①when the tunnel axis is located directly below the "discontinuous joints", as shown in Fig. 4a, it is defined as "odd"; ②when the tunnel axis is located directly below the centre of the pipeline section, as shown in Fig. 4b, it is defined as "even".

**Figure 5:**
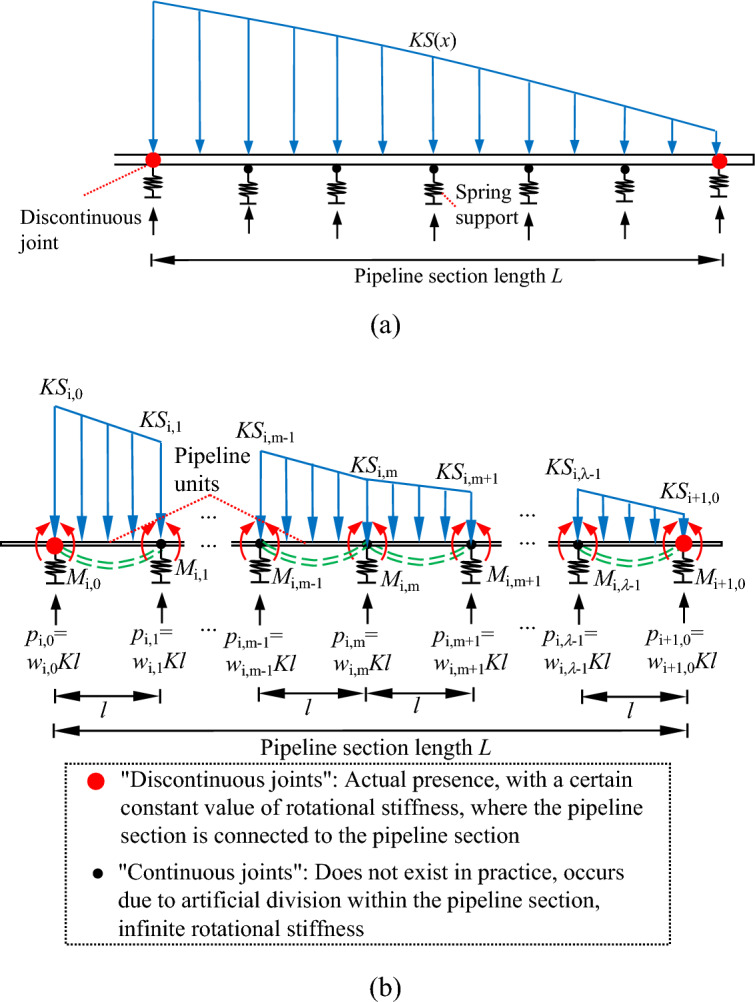
Artificial division of pipeline sections for: (**a**) determining *l*; (**b**) determining the basic system. Note: in the index (*i*, *m*), the first number* i* indicates the number of the pipeline section, and the second number *m* indicates the number of the joint divided within the pipeline section. Both *i* and *m* are numbered from 0 in this study.

In this study, a pipeline section is selected and its division idea is explained in detail as follows. Based on the rigid bar method, the support of the soil on the pipeline is replaced by a finite number of spring supports, as shown in Fig. 5a, which simplifies the problem to a finite statically indeterminate problem, and the spacing of the spring supports is determined according to the accuracy requirements. As shown in Fig. 5b, a simply supported beam is used as the basic system; the pipeline is divided into equal finite parts called pipeline units; the connection points between the pipeline units and the pipeline units are called joints and the interior of the pipeline units is continuous and homogeneous; the stiffness of the spring support is *Kl* (*K* is the modified soil modulus of elasticity and *l* is the length of the pipeline unit). Two types of joints will occur when dividing discontinuous pipelines: ①"continuous joint", this joint is continuous in the pipeline section, and the rotational stiffness is infinite; ②"discontinuous joint", the joint is located in the pipeline section and the pipeline section connected, and the rotational stiffness is related to the way the pipeline section is connected. In the calculation, the length of both joints is ignored and the joint is considered to be a point. The "continuous joint" is regarded as an elastic hinge, the bending moment on both sides of the elastic hinge is the same, and when the rotational stiffness of the "continuous joint" is infinite, the rotation angle of the joint is 0; the "discontinuous joint" is regarded as an elastic hinge, the bending moment on both sides of the elastic hinge is the same, and the rotational stiffness of the "discontinuous joint" is related to the connection method between the pipeline sections, and the rotation angle of the joint is not 0. Herein, the rotational stiffness *β* of the "continuous joint" is infinite and the rotational stiffness *β*_0_ of the "discontinuous joint" is a constant value.

The pipeline section is divided as shown in Fig. 5b and only the schematic diagram of the pipeline section *i* is drawn considering the space limitation. The relationship between the support reaction force and displacement at the joint locations is *p*_i,m_ = *w*_i,m_*Kl*.

## Formulation of the pipeline deflection

Only the situation where the pipeline and the tunnel are perpendicular to each other is considered. Based on the rigid bar method, a simply supported beam is used as the basic system. The support of the pipeline by the soil is considered as a spring support concentrated at the joint. The relationship between the spring support reaction force and the settlement of the joint is shown in Fig. 5b.

**Figure 6:**
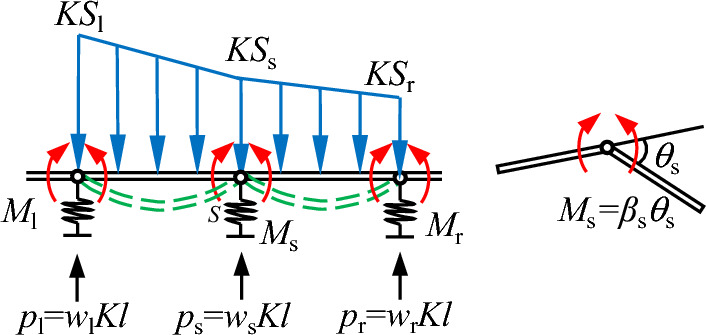
Schematic diagram of general formula calculation.

The aim of this study is to solve for the vertical displacement and rotation angle of the joint. Firstly, the general formula for the vertical displacement *w*_s_ and the general formula for the bending moment *M*_s_ of each joint are established in this study, as shown in Fig. 6. The joint *s* is selected and the process of solving for the support reaction force is explained in detail: The authors select the simply supported beam on the left side of the joint, establish Eq. (4a) according to moment balance and solve for the support reaction force *p*_s_^l^; select the simply supported beam on the right side of the joint, establish Eq. (4b) according to moment balance and solve for the support reaction force *p*_s_^r^; solve for the total support reaction force of the joint according to Eq. (4c), converting the support reaction force to the vertical displacement of the joint Eq. (4e).

4a $$p_{{\text{s}}}^{{\text{l}}} l + M_{{\text{l}}} = M_{{\text{s}}} + Kl^{2} \left( {S_{{\text{l}}} + 2S_{{\text{s}}} } \right)/6$$

4b $$p_{{\text{s}}}^{{\text{r}}} l + M_{{\text{r}}} = M_{{\text{s}}} + Kl^{2} \left( {2S_{{\text{s}}} + S_{{\text{r}}} } \right)/6$$

4c $$p_{{\text{s}}}^{{\text{r}}} + p_{{\text{s}}}^{{\text{l}}} = p_{{\text{s}}}$$

4d $$p_{{\text{s}}} l + M_{{\text{l}}} - 2M_{{\text{s}}} + M_{{\text{r}}} = Kl^{2} \left( {S_{{\text{l}}} + 4S_{{\text{s}}} + S_{{\text{r}}} } \right)/6$$

4e $$w_{{\text{s}}} + \left( {M_{{\text{l}}} - 2M_{{\text{s}}} + M_{{\text{r}}} } \right)/\left( {Kl^{2} } \right) = \left( {S_{{\text{l}}} + 4S_{{\text{s}}} + S_{{\text{r}}} } \right)/6$$

5 $$K = \frac{1.30E}{{1 - v^{2} }}\sqrt[{12}]{{\frac{{Ed^{4} }}{{E_{{\text{p}}} I_{{\text{p}}} }}}}$$

The rotation angle of the joint is solved according to Fig. 6. Through the knowledge of structural mechanics, the rotation angle at the beam end of the simply supported beam due to distributed loads and bending moments is calculated, the angle of rotation θ_s_ of the joint is solved for and calculated as shown in Eq. (6a). After multiplying both sides of Eq. (6a) by the rotational stiffness of the joint *β*_s_, it is then simplified and collapsed into Eq. (6c).

6a $$\theta_{{\text{s}}} = \frac{{w_{{\text{l}}} - 2w_{{\text{s}}} + w_{{\text{r}}} }}{l} + \frac{{KS_{{\text{r}}} l^{3} }}{{24E_{{\text{p}}} I_{{\text{p}}} }} + \frac{{K\left( {S_{{\text{s}}} - S_{{\text{r}}} } \right)l^{3} }}{{45E_{{\text{p}}} I_{{\text{p}}} }} - \frac{{M_{{\text{s}}} l}}{{3E_{{\text{p}}} I_{{\text{p}}} }} - \frac{{M_{{\text{r}}} l}}{{6E_{{\text{p}}} I_{{\text{p}}} }} + \frac{{KS_{{\text{s}}} l^{3} }}{{24E_{{\text{p}}} I_{{\text{p}}} }} + \frac{{7K\left( {S_{{\text{l}}} - S_{{\text{s}}} } \right)l^{3} }}{{360E_{{\text{p}}} I_{{\text{p}}} }} - \frac{{M_{{\text{s}}} l}}{{3E_{{\text{p}}} I_{{\text{p}}} }} - \frac{{M_{{\text{l}}} l}}{{6E_{{\text{p}}} I_{{\text{p}}} }}$$

6b $$M_{{\text{s}}} = \beta_{{\text{s}}} \frac{{w_{{\text{l}}} - 2w_{{\text{s}}} + w_{{\text{r}}} }}{l} + \frac{{\beta_{{\text{s}}} Kl^{3} }}{{360E_{{\text{p}}} I_{{\text{p}}} }}\left( {7S_{{{\text{l}}}} + 16S_{{\text{s}}} + 7S_{{\text{r}}} } \right) - \frac{{\beta_{{\text{s}}} l}}{{6E_{{\text{p}}} I_{{\text{p}}} }}\left( {2M_{{\text{l}}} + 4M_{{\text{s}}} + 2M_{{\text{r}}} } \right)$$

6c $$w_{{\text{l}}} - 2w_{{\text{s}}} + w_{{\text{r}}} = \frac{{l^{2} }}{{6E_{{\text{p}}} I_{{\text{p}}} }}\left[ {M_{{\text{l}}} + \left( {4 + \frac{{6E_{{\text{p}}} I_{{\text{p}}} }}{{\beta_{{\text{s}}} l}}} \right)M_{{\text{s}}} + M_{{\text{r}}} } \right] - Kl^{4} \frac{{7S_{{\text{l}}} + 16S_{{\text{s}}} + 7S_{{\text{r}}} }}{{360E_{{\text{p}}} I_{{\text{p}}} }}$$

where *p*_s_^l^ and *p*_s_^r^ are the support reaction forces, which are provided by the simply supported beam to the left of the joint and the simply supported beam to the right of the joint; θ_s_ is the relative rotation angle of the two sides of the fitting, positive with the right pipeline unit of the fitting rotating clockwise with respect to the left pipeline unit; *M*_l_, *M*_s_ and *M*_r_ are the bending moments of the joint, the positive and negative of which are the same as the angle of rotation of the joint; *S*_l_, *S*_s_ and *S*_r_ are the vertical settlements of the soil at the joints; *E* is soil elastic modulus; *v* is the soil Poisson's ratio; *E*_p_*I*_p_ is the bending stiffness of the pipeline; *β*_s_ is the rotational stiffness of the joint; *K* is the modified soil modulus of elasticity, which can be calculated by the method proposed by Vesic et al.^24^ and modified by Attewell et al.^1^, as shown in Eq. (5).

Continuous pipelines have only one type of joint, i.e. "continuous joint", but discontinuous pipelines have two types of joint, i.e. "discontinuous joint" and "continuous joint". The rotational stiffness of "discontinuous joints" is represented by *β*_s_ = *β*_0_ and that of "continuous joints" by *β*_s_ = *β*. For "discontinuous joints", there are two limit states for the value of the rotational stiffness: ①When *β*_0_ → 0, the pipeline is hinged in this location; ②When *β*_0_ → ∞, the pipeline is continuous in this position.

In this study, the two main types of analysis are "odd" and "even". Due to the symmetry of the pipeline deformation, only the right half of the tunnel axis is taken for analysis.

### "Odd"

**Figure 7:**
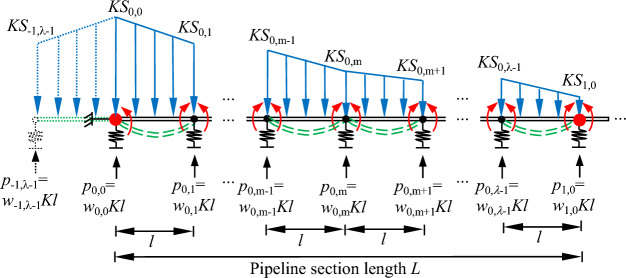
Calculation model of discontinuous pipelines in the "odd" case.

The tunnel axis is located directly below the "discontinuous joint". Due to space constraints, only the first pipeline section directly above the tunnel axis to the right is drawn, as shown in Fig. 7. In the "odd" case *n* pipeline sections are taken for study, the pipeline section length is *L*. Each pipeline section is divided into an even number of parts *λ*, and the length of the pipeline unit is *l* = *L*/*λ*. Calculate the total length *nL*, with a total of *nλ* + 1 joints, of which *n* + 1 are "discontinuous joints" and *n* (*λ* − 1) are "continuous joints".

The pipeline units are considered as simply supported beams with bending moments at the joint locations. The additional load acting on the pipeline by the soil is considered as a linear distributed load on each pipeline unit.

For each joint the equations containing *w*_s_ and *M*_s_ can be established according to Eqs. (4e) and (6b). It should be noted that there are two different types of joints: ①"discontinuous joints" and ②"continuous joints".

For ①"discontinuous joints":

7a $$w_{{\text{i,0}}} + \left( {M_{{{\text{i}} - 1,\lambda - 1}} - 2M_{{\text{i,0}}} + M_{{\text{i,1}}} } \right)/\left( {Kl^{2} } \right) = \left( {S_{{{\text{i - 1,}}\lambda { - 1}}} + 4S_{{\text{i,0}}} + S_{{\text{i,1}}} } \right)/6$$

7b $$w_{{{\text{i - 1,}}\lambda { - 1}}} - 2w_{{\text{i,0}}} + w_{{\text{i,1}}} = \frac{{l^{2} }}{{6E_{{\text{p}}} I_{{\text{p}}} }}\left[ {M_{{{\text{i - 1,}}\lambda { - 1}}} + \left( {4 + \frac{{6E_{{\text{p}}} I_{{\text{p}}} }}{{\beta_{0} l}}} \right)M_{{\text{i,0}}} + M_{{\text{i,1}}} } \right] - Kl^{4} \frac{{7S_{{{\text{i - 1,}}\lambda { - 1}}} + 16S_{{\text{i,0}}} + 7S_{{\text{i,1}}} }}{{360E_{{\text{p}}} I_{{\text{p}}} }}$$

For ②"continuous joints":

8a $$w_{{\text{i,m}}} + \left( {M_{{\text{i,m - 1}}} - 2M_{{\text{i,m}}} + M_{{\text{i,m + 1}}} } \right)/\left( {Kl^{2} } \right) = \left( {S_{{\text{i,m - 1}}} + 4S_{{\text{i,m}}} + S_{{\text{i,m + 1}}} } \right)/6$$

8b $$w_{{\text{i,m - 1}}} - 2w_{{\text{i,m}}} + w_{{\text{i,m + 1}}} = \frac{{l^{2} }}{{6E_{{\text{p}}} I_{{\text{p}}} }}\left[ {M_{{\text{i,m - 1}}} + \left( {4 + \frac{{6E_{{\text{p}}} I_{{\text{p}}} }}{\beta l}} \right)M_{{\text{i,m}}} + M_{{\text{i,m + 1}}} } \right] - Kl^{4} \frac{{7S_{{\text{i,m - 1}}} + 16S_{{\text{i,m}}} + 7S_{{\text{i,m + 1}}} }}{{360E_{{\text{p}}} I_{{\text{p}}} }}$$

The pipeline deformation is symmetrical about the tunnel axis and boundary conditions can be established as in Eqs. (9a), (9b) and (9c). When the pipeline is long enough and far enough away, it is considered that the pipeline bending moment, pipeline displacement and soil displacement outside the area affected by the tunnel excavation are zero, and boundary conditions Eqs. (9d), (9e) and (9f) can be established.

9a $$M_{{{ - 1,}\lambda { - 1}}} = M_{0,1}$$

9b $$w_{{{ - 1,}\lambda { - 1}}} = w_{0,1}$$

9c $$S_{{{ - 1,}\lambda { - 1}}} = S_{0,1}$$

9d $$M_{{\text{n,1}}} = 0$$

9e $$w_{{\text{n,1}}} = 0$$

9f $$S_{{\text{n,1}}} = 0$$

In the above equations,* w*_i, m_ and *M*_i, m_ are unknown quantities, a total of 2*nλ* + 6 unknowns, in which 2*nλ* + 2 equations can be created for each joint in the format of Eqs. (7a), (7b), (8a) and (8b), combined with the boundary conditions Eqs. (9a), (9b), (9c), (9d), (9e) and (9f). The support reaction forces and the bending moments of the joints can be represented in matrix form after collation, as shown in Eqs. (10a), (10b), (10c) and (10d). The bending moment of each joint is calculated from Eq. (10d), and then the rotation angle of each joint is calculated from Eq. (10e).

10a $$\left\{ w \right\} = \left[ A \right]\left\{ S \right\} - \left[ B \right]\left\{ M \right\}$$

10b $$\left[ C \right]\left\{ w \right\} = \left[ D \right]\left\{ M \right\} - \left[ E \right]\left\{ S \right\}$$

10c $$\left[ {\left[ B \right]^{ - 1} + \left[ D \right]^{ - 1} \left[ C \right]} \right]\left\{ w \right\} = \left[ {\left[ B \right]^{ - 1} \left[ A \right] - \left[ D \right]^{ - 1} \left[ E \right]} \right]\left\{ S \right\}$$

10d $$\left[ {\left[ C \right]\left[ B \right] + \left[ D \right]} \right]\left\{ M \right\} = \left[ {\left[ C \right]\left[ A \right] + \left[ E \right]} \right]\left\{ S \right\}$$

10e $$M_{{\text{s}}} = \beta_{{\text{s}}} \theta_{{\text{s}}}$$

By coupling Eqs. (7a), (7b), (8a), (8b), (9a), (9b), (9c), (9d), (9e) and (9f). The matrices and vectors in Eqs. (10c) and (10d) can be represented in detail as follows.

11a $$\{ w\} = \{ w_{0,0} w_{0,1} \ldots w_{0,m} \ldots w_{{{0,}\lambda { - 1}}} w_{1,0} \ldots w_{{\text{i,0}}} w_{{\text{i,1}}} \ldots w_{{\text{i,m}}} { } \ldots w_{{{\text{i,}}\lambda { - 1}}} w_{{\text{i + 1,0}}} \ldots w_{{\text{n - 1,0}}} w_{{\text{n - 1,1}}} \ldots w _{{{\text{n}} - 1,{\text{m}}}} \ldots w_{{{\text{n - 1,}}\lambda { - 1}}} w_{{\text{n,0}}} \}^{T}$$

11b $$\{ M\} = \{ M_{0,0} M_{0,1} \ldots M_{0,m} \ldots M_{{{0,}\lambda { - 1}}} M_{1,0} \ldots M_{{\text{i,0}}} M_{{\text{i,1}}} \ldots M_{{\text{i,m}}} { } \ldots M_{{{\text{i,}}\lambda { - 1}}} M_{{\text{i + 1,0}}} \ldots M_{{\text{n - 1,0}}} M_{{\text{n - 1,1}}} \ldots M _{{{\text{n}} - 1,{\text{m}}}} \ldots M_{{{\text{n - 1,}}\lambda { - 1}}} M_{{\text{n,0}}} \}^{T}$$

11c $$\{ S\} = \{ S_{0,0} S_{0,1} \ldots S_{0,m} \ldots S_{{{0,}\lambda { - 1}}} S_{1,0} \ldots S_{{\text{i,0}}} S_{{\text{i,1}}} \ldots S_{{\text{i,m}}} { } \ldots S_{{{\text{i,}}\lambda { - 1}}} S_{{\text{i + 1,0}}} \ldots S_{{\text{n - 1,0}}} S_{{\text{n - 1,1}}} \ldots S _{{{\text{n}} - 1,{\text{m}}}} \ldots S_{{{\text{n - 1,}}\lambda { - 1}}} S_{{\text{n,0}}} \}^{T}$$

11d $$S_{{\text{i,m}}} = S\left[ {\left( {i\lambda + m} \right)l} \right]$$

12a $$\left[ A \right] = \frac{1}{6}\left[ {\begin{array}{*{20}c} 4 & 2 & 0 & \cdots & 0 \\ 1 & 4 & 1 & \ddots & \vdots \\ 0 & \ddots & \ddots & \ddots & 0 \\ \vdots & \ddots & 1 & 4 & 1 \\ 0 & \cdots & 0 & 1 & 4 \\ \end{array} } \right]$$

12b $$\left[ B \right] = \frac{1}{{Kl^{2} }}\left[ {\begin{array}{*{20}c} { - 2} & 2 & 0 & \cdots & 0 \\ 1 & { - 2} & 1 & \ddots & \vdots \\ 0 & \ddots & \ddots & \ddots & 0 \\ \vdots & \ddots & 1 & { - 2} & 1 \\ 0 & \cdots & 0 & 1 & { - 2} \\ \end{array} } \right]$$

12c $$\left[ C \right] = \left[ {\begin{array}{*{20}c} { - 2} & 2 & 0 & \cdots & 0 \\ 1 & { - 2} & 1 & \ddots & \vdots \\ 0 & \ddots & \ddots & \ddots & 0 \\ \vdots & \ddots & 1 & { - 2} & 1 \\ 0 & \cdots & 0 & 1 & { - 2} \\ \end{array} } \right]$$

12d $$\left[ D \right] = \frac{{l^{2} }}{{6E_{{\text{p}}} I_{{\text{p}}} }}\left[ {\begin{array}{*{20}c} {\left[ D \right]_{0} } \\ \vdots \\ {\left[ D \right]_{{\text{i}}} } \\ \vdots \\ {\left[ D \right]_{{\text{n - 1}}} } \\ \end{array} } \right]$$

12e $$\left[ E \right] = \frac{{Kl^{4} }}{{360E_{{\text{p}}} I_{{\text{p}}} }}\left[ {\begin{array}{*{20}c} {16} & {14} & 0 & \cdots & 0 \\ 7 & {16} & 7 & \ddots & \vdots \\ 0 & \ddots & \ddots & \ddots & 0 \\ \vdots & \ddots & 7 & {16} & 7 \\ 0 & \cdots & 0 & 7 & {16} \\ \end{array} } \right]$$

The matrix [*D*] is represented in detail as Eqs. (12f), (12g) and (12h).

12f $$\left[ D \right]_{0} = \left[ {\left[ {\begin{array}{*{20}c} {\beta_{{\text{b}}} } & 2 & {} & {} & {} & {} & {} & {} \\ 1 & {\beta_{{\text{c}}} } & 1 & {} & {} & {} & {} & {} \\ {} & 1 & {\beta_{{\text{c}}} } & 1 & {} & {} & {} & {} \\ {} & {} & \ddots & \ddots & \ddots & {} & {} & {} \\ {} & {} & {} & 1 & {\beta_{{\text{c}}} } & 1 & {} & {} \\ {} & {} & {} & {} & \ddots & \ddots & \ddots & {} \\ {} & {} & {} & {} & {} & 1 & {\beta_{{\text{c}}} } & 1 \\ \end{array} } \right]_{{\lambda \times \left( {\lambda { + 1}} \right)}} \left[ 0 \right]_{{\lambda \times \left( {{\text{n}}\lambda { - }\lambda } \right)}} } \right]$$

12g $$\left[ D \right]_{{\text{i}}} = \left[ {\left[ 0 \right]_{{\lambda \times \left( {{\text{i}}\lambda { - 1}} \right)}} \left[ {\begin{array}{*{20}c} 1 & {\beta_{{\text{b}}} } & 1 & {} & {} & {} & {} & {} & {} \\ {} & 1 & {\beta_{{\text{c}}} } & 1 & {} & {} & {} & {} & {} \\ {} & {} & 1 & {\beta_{{\text{c}}} } & 1 & {} & {} & {} & {} \\ {} & {} & {} & \ddots & \ddots & \ddots & {} & {} & {} \\ {} & {} & {} & {} & 1 & {\beta_{{\text{c}}} } & 1 & {} & {} \\ {} & {} & {} & {} & {} & \ddots & \ddots & \ddots & {} \\ {} & {} & {} & {} & {} & {} & 1 & {\beta_{{\text{c}}} } & 1 \\ \end{array} } \right]_{{\lambda \times \left( {\lambda { + 2}} \right)}} \left[ 0 \right]_{{\lambda \times \left( {\lambda \left( {\text{n - i - 1}} \right)} \right)}} } \right]$$

12h $$\left[ D \right]_{{\text{n - 1}}} = \left[ {\left[ 0 \right]_{{\left( {\lambda { + 1}} \right) \times \left( {\left( {\text{n - 1}} \right)\lambda { - 1}} \right)}} \left[ {\begin{array}{*{20}c} 1 & {\beta_{{\text{b}}} } & 1 & {} & {} & {} & {} & {} & {} \\ {} & 1 & {\beta_{{\text{c}}} } & 1 & {} & {} & {} & {} & {} \\ {} & {} & 1 & {\beta_{{\text{c}}} } & 1 & {} & {} & {} & {} \\ {} & {} & {} & \ddots & \ddots & \ddots & {} & {} & {} \\ {} & {} & {} & {} & 1 & {\beta_{{\text{c}}} } & 1 & {} & {} \\ {} & {} & {} & {} & {} & \ddots & \ddots & \ddots & {} \\ {} & {} & {} & {} & {} & {} & 1 & {\beta_{{\text{c}}} } & 1 \\ {} & {} & {} & {} & {} & {} & {} & 1 & {\beta_{{\text{b}}} } \\ \end{array} } \right]_{{\left( {\lambda { + 1}} \right) \times \left( {\lambda { + 2}} \right)}} } \right]$$

in which *β*_c_ = 4 + 6*E*_p_*I*_p_/(*lβ*), *β*_b_ = 4 + 6*E*_p_*I*_p_/(*lβ*_0_).

Equations (12a), (12b), (12c), (12d), (12e), (12f), (12g) and (12h) are substituted into Eqs. (10c) and (10d), and the deflection of the discontinuous pipeline and the bending moment of the joint in the "odd" case can be solved for, followed by the angle of rotation of the joint.

### "Even"

**Figure 8:**
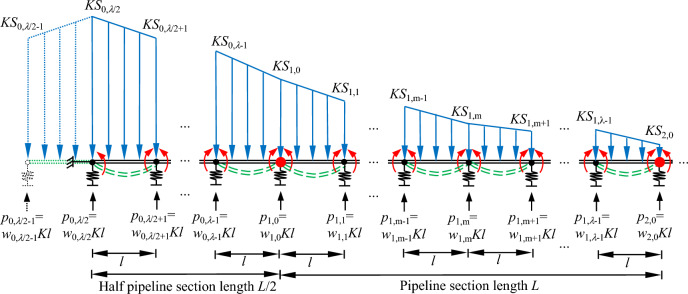
Calculation model of discontinuous pipelines in the "even" case.

The tunnel axis is located directly below the centre of the pipeline section, in view of space constraints, only one and a half pipeline sections directly above the tunnel axis are drawn, as shown in Fig. 8. Half of the pipeline section, and the *n* pipeline sections to it's right are analysed. Each pipeline section is divided into an even number of parts *λ*, the pipeline section length is *L* and the length of pipeline unit is *l* = *L*/*λ*; the total calculated length is taken as *nL* + 0.5*L*, a total of (*n* + 0.5)*λ* + 1 joints, of which "discontinuous joints" *n* + 1, and "continuous joints" *n*(*λ* − 1) + 0.5*λ*.

The calculation process in the "odd" and "even" cases is the same; the difference is that in the "even" case the tunnel axis is located directly below the centre of the pipeline section, whereas in the "odd" case the tunnel axis is located directly below the "discontinuous joint". The same Eqs. (4e), (6b), (7a), (7b), (8a) and (8b) can be established in the "even" case.

The boundary conditions established in the "even" case are Eqs. (13a), (13b), (13c), (13d), (13e) and (13f).

13a $$M_{{{0,}\lambda {/2 - 1}}} = M_{{0,\lambda {/2 + }1}}$$

13b $$w_{{{0,}\lambda {/2 - 1}}} = w_{{0,\lambda {/2 + }1}}$$

13c $$S_{{{0,}\lambda {/2 - 1}}} = S_{{0,\lambda {/2 + }1}}$$

13d $$M_{{\text{n + 1,1}}} = 0$$

13e $$w_{{\text{n + 1,1}}} = 0$$

13f $$S_{{\text{n + 1,1}}} = 0$$

In the "even" case, *w*_i, m_ and *M*_i, m_ are unknown quantities, a total of 2*nλ* + *λ* + 6. 2*nλ* + *λ* + 2 equations can be established according to Eqs. (7a), (7b), (8a) and (8b), and then combined with the boundary conditions Eqs. (13a), (13b), (13c), (13d), (13e) and (13f). The vertical displacements and bending moments of the joints can be represented in matrix form after collation, as shown in Eqs. (14a), (14b), (14c) and (14d). The bending moment of each joint is calculated from Eq. (14d), and then the rotation angle of each joint is calculated from Eq. (14e).

14a $$\left\{ w \right\} = \left[ A \right]^{\prime } \left\{ S \right\} - \left[ B \right]^{\prime } \left\{ M \right\}$$

14b $$\left[ C \right]^{\prime } \left\{ w \right\} = \left[ D \right]^{\prime } \left\{ M \right\} - \left[ E \right]^{\prime } \left\{ S \right\}$$

14c $$\left[ {\left[ B \right]^{\prime - 1} + \left[ D \right]^{\prime - 1} \left[ C \right]^{\prime } } \right]\left\{ w \right\} = \left[ {\left[ B \right]^{\prime - 1} \left[ A \right]^{\prime } - \left[ D \right]^{\prime - 1} \left[ E \right]^{\prime } } \right]\left\{ S \right\}$$

14d $$\left[ {\left[ C \right]^{\prime } \left[ B \right]^{\prime } + \left[ D \right]^{\prime } } \right]\left\{ M \right\} = \left[ {\left[ C \right]^{\prime } \left[ A \right]^{\prime } + \left[ E \right]^{\prime } } \right]\left\{ S \right\}$$

14e $$M_{{\text{s}}} = \beta_{{\text{s}}} \theta_{{\text{s}}}$$

By coupling Eqs. (7a), (7b), (8a) and (8b) and Eqs. (13a), (13b), (13c), (13d), (13e) and (13f), the matrices and vectors in Eqs. (14c) and (14d) can be represented in detail as follows.

15a $$\{ w\} = \{ w_{{{0,}\lambda {/2}}} \ldots w_{{\text{0,m}}} \ldots w_{{{0,}\lambda { - 1}}} w_{{1,0}} w_{{1,1}} \ldots w_{{\text{1,m}}} \ldots w_{{{1,}\lambda { - 1}}} w_{{2,0}} \ldots w_{{\text{i,0}}} w_{{\text{i,1}}} \ldots w_{{\text{i,m}}} \ldots w_{{{\text{i,}}\lambda { - 1}}} w_{{\text{i + 1,0}}} \ldots w_{{\text{n,0}}} w_{{\text{n,1}}} \ldots w_{{\text{n,m}}} \ldots w_{{{\text{n,}}\lambda { - 1}}} w_{{\text{n + 1,0}}} \}^{T}$$

15b $$\{ M\} = \{ M_{{{0,}\lambda {/2}}} \ldots M_{{\text{0,m}}} \ldots M_{{{0,}\lambda { - 1}}} M_{{1,0}} M_{{1,1}} \ldots M_{{\text{1,m}}} \ldots M_{{{1,}\lambda { - 1}}} M_{{2,0}} \ldots M_{{\text{i,0}}} M_{{\text{i,1}}} \ldots M_{{\text{i,m}}} \ldots M_{{{\text{i,}}\lambda { - 1}}} M_{{\text{i + 1,0}}} \ldots M_{{\text{n,0}}} M_{{\text{n,1}}} \ldots M_{{\text{n,m}}} \ldots M_{{{\text{n,}}\lambda { - 1}}} M_{{\text{n + 1,0}}} \}^{T}$$

15c $$\{ S\} = \{ S_{{{0,}\lambda {/2}}} \ldots S_{{\text{0,m}}} \ldots S_{{{0,}\lambda { - 1}}} S_{{1,0}} S_{{1,1}} \ldots S_{{\text{1,m}}} \ldots S_{{{1,}\lambda { - 1}}} S_{{2,0}} \ldots S_{{\text{i,0}}} S_{{\text{i,1}}} \ldots S_{{\text{i,m}}} \ldots S_{{{\text{i,}}\lambda { - 1}}} S_{{\text{i + 1,0}}} \ldots S_{{\text{n,0}}} S_{{\text{n,1}}} \ldots S_{{\text{n,m}}} \ldots S_{{{\text{n,}}\lambda { - 1}}} S_{{\text{n + 1,0}}} \}^{T}$$

15d $$S_{{\text{i,m}}} = S\left[ {\left( {i\lambda - 0.5\lambda + m} \right)l} \right]$$

16a $$\left[ A \right]^{\prime } = \frac{1}{6}\left[ {\begin{array}{*{20}c} 4 & 2 & 0 & \cdots & 0 \\ 1 & 4 & 1 & \ddots & \vdots \\ 0 & \ddots & \ddots & \ddots & 0 \\ \vdots & \ddots & 1 & 4 & 1 \\ 0 & \cdots & 0 & 1 & 4 \\ \end{array} } \right]$$

16b $$\left[ B \right]^{\prime } = \frac{1}{{Kl^{2} }}\left[ {\begin{array}{*{20}c} { - 2} & 2 & 0 & \cdots & 0 \\ 1 & { - 2} & 1 & \ddots & \vdots \\ 0 & \ddots & \ddots & \ddots & 0 \\ \vdots & \ddots & 1 & { - 2} & 1 \\ 0 & \cdots & 0 & 1 & { - 2} \\ \end{array} } \right]$$

16c $$\left[ C \right]^{\prime } = \left[ {\begin{array}{*{20}c} { - 2} & 2 & 0 & \cdots & 0 \\ 1 & { - 2} & 1 & \ddots & \vdots \\ 0 & \ddots & \ddots & \ddots & 0 \\ \vdots & \ddots & 1 & { - 2} & 1 \\ 0 & \cdots & 0 & 1 & { - 2} \\ \end{array} } \right]$$

16d $$\left[ D \right]^{\prime } = \frac{{l^{2} }}{{6E_{{\text{p}}} I_{{\text{p}}} }}\left[ {\begin{array}{*{20}c} {\left[ D \right]^{\prime }_{0} } \\ \vdots \\ {\left[ D \right]^{\prime }_{{\text{i}}} } \\ \vdots \\ {\left[ D \right]^{\prime }_{{\text{n}}} } \\ \end{array} } \right]$$

16e $$\left[ E \right]^{\prime } = \frac{{Kl^{4} }}{{360E_{{\text{p}}} I_{{\text{p}}} }}\left[ {\begin{array}{*{20}c} {16} & {14} & 0 & \cdots & 0 \\ 7 & {16} & 7 & \ddots & \vdots \\ 0 & \ddots & \ddots & \ddots & 0 \\ \vdots & \ddots & 7 & {16} & 7 \\ 0 & \cdots & 0 & 7 & {16} \\ \end{array} } \right]$$

The matrix [*D*]*'* is represented in detail as Eqs. (16f), (16g) and (16h).

16f $$\left[ D \right]^{\prime }_{0} = \left[ {\left[ {\begin{array}{*{20}c} {\beta_{{\text{c}}} } & 2 & {} & {} & {} & {} & {} \\ 1 & {\beta_{{\text{c}}} } & 1 & {} & {} & {} & {} \\ {} & \ddots & \ddots & \ddots & {} & {} & {} \\ {} & {} & 1 & {\beta_{{\text{c}}} } & 1 & {} & {} \\ {} & {} & {} & \ddots & \ddots & \ddots & {} \\ {} & {} & {} & {} & 1 & {\beta_{{\text{c}}} } & 1 \\ \end{array} } \right]_{{\lambda {/2} \times \left( {\lambda {/2 + 1}} \right)}} \left[ 0 \right]_{{\lambda \times {\text{n}}\lambda }} } \right]$$

16g $$\left[ D \right]^{\prime }_{{\text{i}}} = \left[ {\left[ 0 \right]_{{\lambda \times \left( {{\text{i}}\lambda { - }\lambda {/2 - 1}} \right)}} \left[ {\begin{array}{*{20}c} 1 & {\beta_{{\text{b}}} } & 1 & {} & {} & {} & {} & {} & {} \\ {} & 1 & {\beta_{{\text{c}}} } & 1 & {} & {} & {} & {} & {} \\ {} & {} & 1 & {\beta_{{\text{c}}} } & 1 & {} & {} & {} & {} \\ {} & {} & {} & \ddots & \ddots & \ddots & {} & {} & {} \\ {} & {} & {} & {} & 1 & {\beta_{{\text{c}}} } & 1 & {} & {} \\ {} & {} & {} & {} & {} & \ddots & \ddots & \ddots & {} \\ {} & {} & {} & {} & {} & {} & 1 & {\beta_{{\text{c}}} } & 1 \\ \end{array} } \right]_{{\lambda \times \left( {\lambda { + 2}} \right)}} \left[ 0 \right]_{{\lambda \times \left( {\lambda \left( {\text{n - i}} \right)} \right)}} } \right]$$

16h $$\left[ D \right]^{\prime }_{{\text{n}}} = \left[ {\left[ 0 \right]_{{\left( {\lambda { + 1}} \right) \times \left( {{\text{n}}\lambda - \lambda {/2 - 1}} \right)}} \left[ {\begin{array}{*{20}c} 1 & {\beta_{{\text{b}}} } & 1 & {} & {} & {} & {} & {} & {} \\ {} & 1 & {\beta_{{\text{c}}} } & 1 & {} & {} & {} & {} & {} \\ {} & {} & 1 & {\beta_{{\text{c}}} } & 1 & {} & {} & {} & {} \\ {} & {} & {} & \ddots & \ddots & \ddots & {} & {} & {} \\ {} & {} & {} & {} & 1 & {\beta_{{\text{c}}} } & 1 & {} & {} \\ {} & {} & {} & {} & {} & \ddots & \ddots & \ddots & {} \\ {} & {} & {} & {} & {} & {} & 1 & {\beta_{{\text{c}}} } & 1 \\ {} & {} & {} & {} & {} & {} & {} & 1 & {\beta_{{\text{b}}} } \\ \end{array} } \right]_{{\left( {\lambda + 1} \right) \times \left( {\lambda + 2} \right)}} } \right]$$

Equations (16a), (16b), (16c), (16d), (16e), (16f), (16g) and (16h) are substituted into Eqs. (14c) and (14d), and the deflection of the discontinuous pipeline and the bending moment of the joint in the "even" case can be solved for, followed by the angle of rotation of the joint.

In view of the above, the calculation of the pipeline deformation for the two different cases is completed. If the deformation of a continuous pipeline needs to be solved for, simply replace *β*_0_ with *β*.

### Calculation procedure

**Figure 9:**
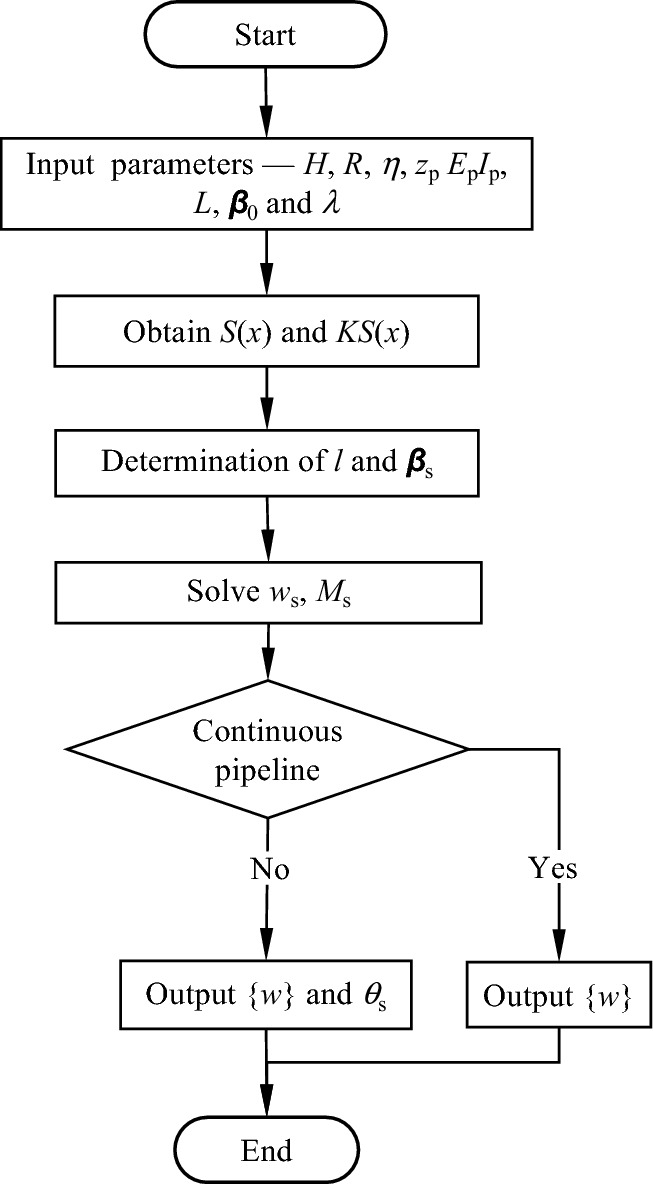
Flow chart for calculation.

Figure 9 outlines the calculation procedure for pipeline deformation and the main calculation procedure for pipeline deformation, described in detail as follows:

1. Calculation of the greenfield displacement *S*(*x*) and calculation of the additional load *KS*(*x*) acting on the pipeline;
2. Calculate the length of the pipeline unit *l*, and determine the rotational stiffness *β*_s_ of each joint (*β* for "continuous joints" and *β*_0_ for "discontinuous joints");
3. Based on the knowledge of structural mechanics, the equations for each joint containing *w*_s_ and *M*_s_ are established according to Eqs. (4e) and (6c);
4. Combining the boundary conditions, calculate the bending moment of the joint and the deflection of the pipeline. If the pipeline is discontinuous, output the pipeline deflection {*w*} and the joint rotation angle θ_s_. If the pipeline is continuous, output the pipeline deflection {*w*}.

## Example verification

### Comparison with field measured data for continuous pipelines

Ma^25^ provided the measured data of shield tunnel excavation in a certain section of the Shenzhen metro project. This section of the tunnel is located in gravelly clay and sandy clay, which is a typical soil tunnel, the pipeline is perpendicular to the tunnel. The relevant calculation parameters for the engineering example are shown in Table 1.

**Table 1:** Calculation parameters.

*H*(m)	*R*(m)	*z*_p_(m)	*d*(m)	*E*_p_*I*_p_(N·m)	*E*(N/m^2^)	*v*	*η*
14.4	3	8.7	3	2.819 × 10^10^	8.2 × 10^6^	0.3	0.77%

The calculated length of the pipeline unit is *l* = 0.2 m. This project was used to determine the specific values of the rotational stiffness of the "continuous joint" in the calculation.

In performing the actual calculation, it is not possible to substitute *β* = ∞ into the calculation, but by taking a certain larger value into the calculation, the accuracy of the calculation can also be satisfied.

To ensure that the rotational stiffness of the "continuous joint" is sufficiently large, it is recommended that the rotational stiffness of the "continuous joint" is calculated using Eq. (17), by taking the larger value to indicate that the joint is continuous.

17 $$\beta = uE_{{\text{p}}} I_{{\text{p}}} /l$$

where *u* is the calculation parameter.

**Figure 10:**
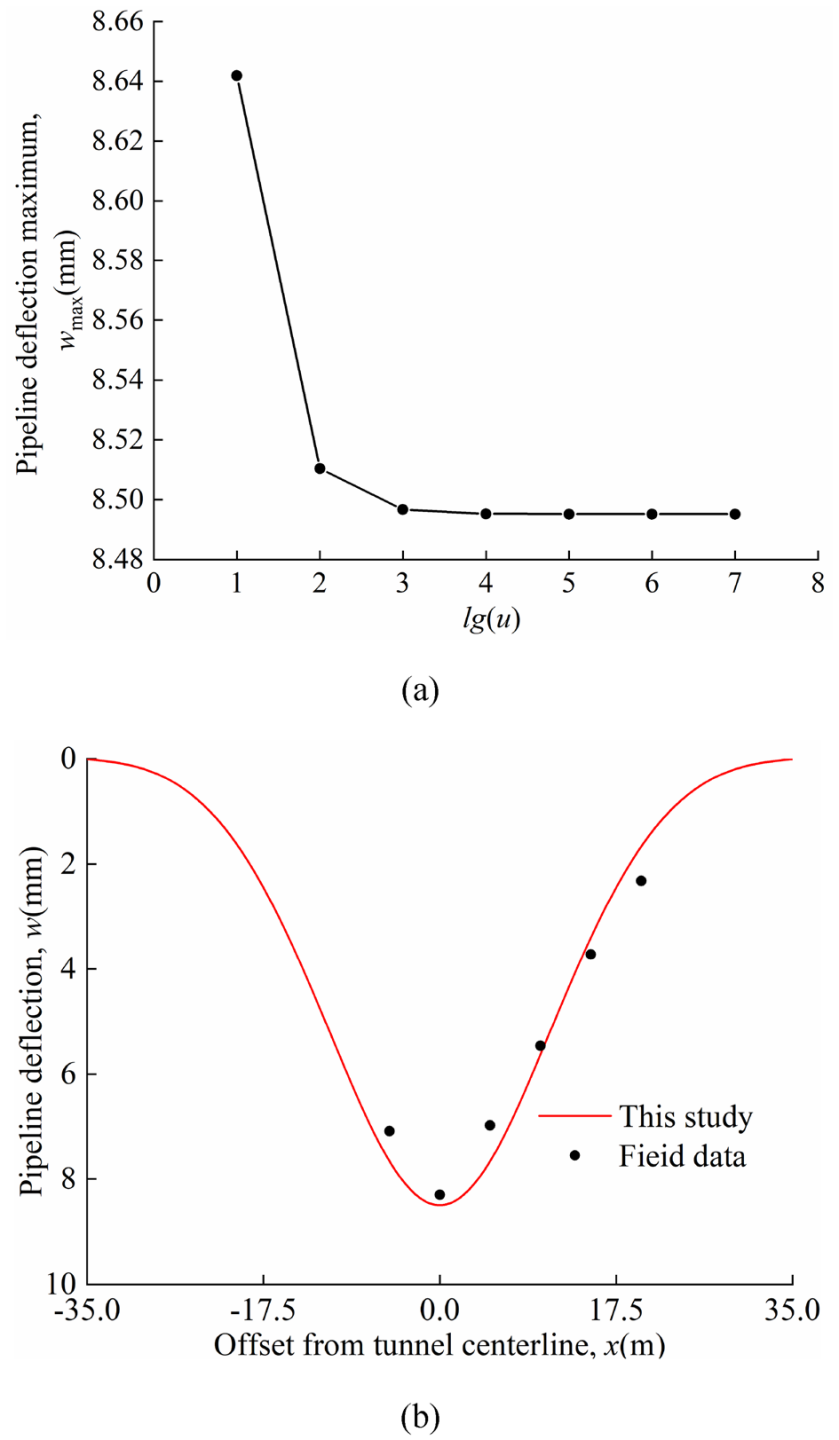
Comparison with field data: (**a**) variation of the maximum deflection of the pipeline with *β*; (**b**) calculated and measured values.

By changing the value of *u*, the relationship between the maximum deflection value of the pipeline and the change of *lg*(*u*) is obtained as shown in Fig. 10a, from which it can be found that as the value of *lg*(*u*) increases, the maximum deflection value of the pipeline gradually decreases. At *lg*(*u*) = 4, 5 and 6, the calculated maximum values of pipeline deflection are 8.4953 mm, 8.4952 mm and 8.4952 mm respectively, which shows that there is basically no change in the maximum value of pipeline deflection after *lg*(*u*) = 5. Therefore, this study determined that *u* = 10^5^, i.e. the rotational stiffness of the "continuous joint" *β* = 10^5^*E*_p_*I*_p_/*l*.

Figure 10b shows a comparison of the calculated results with the field data, from which it can be seen that the calculated results are in good agreement with the field data. The deviation between the maximum pipeline deflection calculated by this study method and the field value is 2.35%. The correctness of this study in solving for continuous pipeline deformation is verified.

This study defines the deviation between predicted values and field values as: deviation = (predicted value- field value)/field value.

### Comparison with the centrifuge test for discontinuous pipelines

Vorster^26^ measured the vertical deformation of the pipeline caused by tunnel excavation under the condition of centrifugal acceleration of 75 g. The details of the soil body, the jointed model pipelines and the test procedure are described in Vorster^26^. The relevant calculated parameters for the centrifuge test are shown in Table 2.

**Table 2:** Calculation parameters.

Test	*H*(m)	*R*(m)	*z*_p_(m)	*d*(m)	*E*_p_*I*_p_(N·m^2^)	*E*(N/m^2^)	*v*	*η*	*β*_0_(N·m)	*L*	
1	11.25	2.25	4.165	1.19	3.36 × 10^9^	19.52 × 10^6^	0.4	0.3%	4.47 × 10^4^	5.34	Odd
2	11.25	2.25	4.165	1.19	3.36 × 10^9^	19.52 × 10^6^	0.4	2%	4.47 × 10^4^	5.34	
3	11.25	2.25	4.165	1.19	3.36 × 10^9^	19.52 × 10^6^	0.4	0.3%	4.47 × 10^4^	5.34	Even
4	11.25	2.25	4.165	1.19	3.36 × 10^9^	19.52 × 10^6^	0.4	2%	4.47 × 10^4^	5.34	

**Figure 11:**
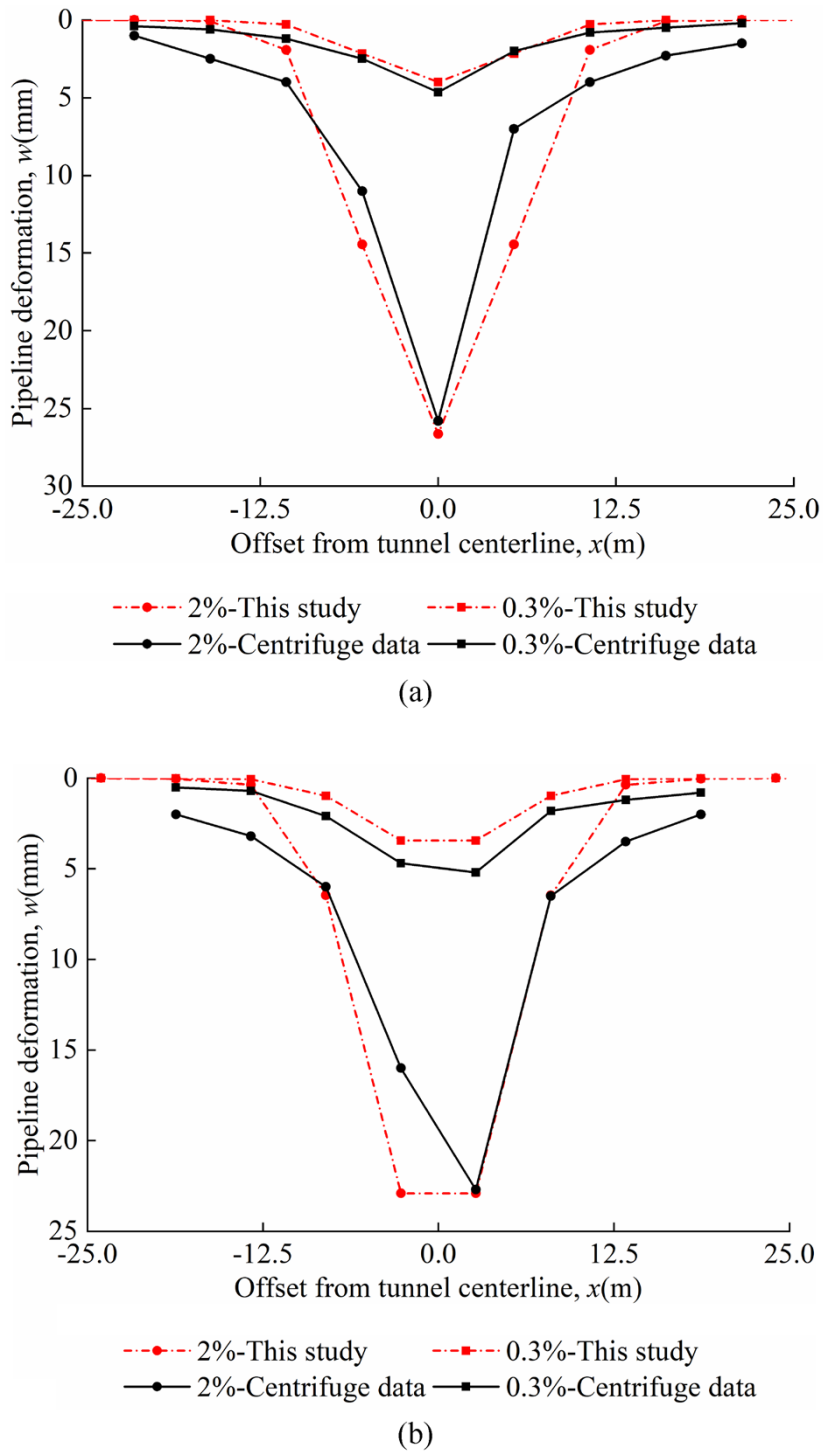
Comparison with centrifuge experiment for: (**a**) odd; (**b**) even.

Figures 11a and b show a comparison of the calculated results of the method proposed in this study with the experimental data. For test 1, the deviation between the maximum pipeline deflection calculated by this study method and the experimental value is − 6.12%. For test 2, the deviation between the maximum pipeline deflection calculated by this study method and the experimental value is 4.42%. For test 3, the deviation between the maximum pipeline deflection calculated by this study method and the experimental value is − 31.27%. For test 4, the deviation between the maximum pipeline deflection calculated by this study method and the experimental value is 0.88%. The maximum deflection value in the "odd" case is found to be greater than the maximum deflection value in the "even" case. The calculated results are in good agreement with the experimental data, which verifies the correctness of the method.

### Comparison with field measured data for discontinuous pipelines

Sun et al.^27^ carried out deflection monitoring of gas pipelines affected by shield tunnel excavation. Based on the information of this project, provided by Zhang et al.^11^ and Sun et al.^27^, the relevant calculation parameters for the engineering example are shown in Table 3.

**Table 3:** Calculation parameters.

*H*(m)	*R*(m)	*z*_p_(m)	*d*(m)	*E*_p_*I*_p_(N·m^2^)	*E*(N/m^2^)	*v*	*η*	*β*_0_(N·m)	*L*	
15.38	3.1	0.9	0.326	1.52 × 10^7^	7.89 × 10^6^	0.3	0.5%	5.18 × 10^5^	5	Even

**Figure 12:**
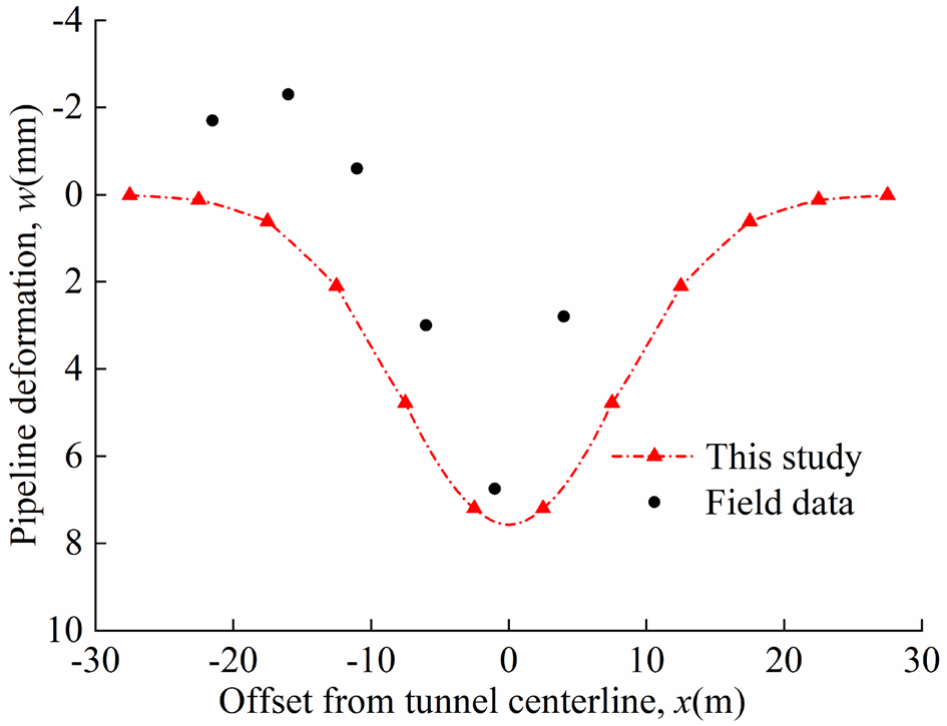
Comparison with field data.

Figure 12 shows a comparison of the calculated results of the method proposed in this study with the field data. The deviation between the maximum pipeline deflection calculated by this study method and the field value is 11.43%. During the tunnel excavation, the soil was uplifted, which in turn caused the pipeline to bulge, but this phenomenon was not taken into account in Peck's empirical equation, thus making the calculated results deviate from the field data at some locations. On the whole, the calculated values are in good agreement with the field data, which confirms the correctness of the method.

## Parametric studies

A parametric study is carried out with reference to the centrifuge experiments of Vorster^26^ where two cases of "odd" and "even" are analyzed. This paper focuses on the effect of volume loss, ratio of pipeline section length to inflection point of soil settlement curve, the rotational stiffness of the "discontinuous joint" and soil elastic modulus on the deflection of the pipeline and the maximum angle of rotation of the joint. For comparison purposes, the authors have taken absolute values for the joint rotation angles.

### Volume loss

Different volume losses are analysed *η* = 1%, 2%, 3%. The other parameters are consistent with those of the model centrifuge tests carried out by Vorster^26^. The pipeline deflection and maximum rotation angle of joints in "odd" and "even" cases have been discussed.

**Figure 13:**
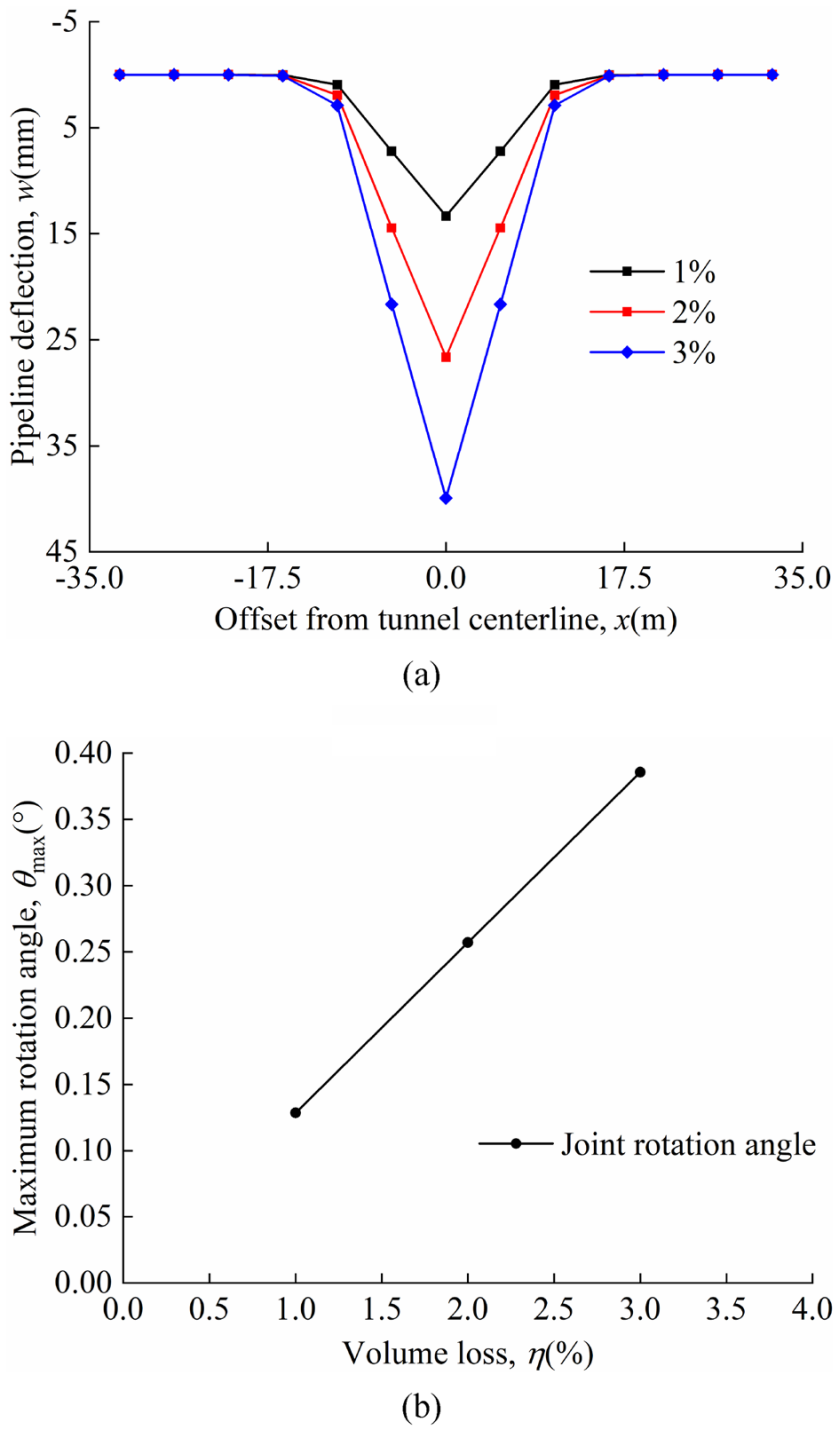
Different volume losses in the "odd" case: (**a**) pipeline deflection; (**b**) variation of maximum rotation angle with *η*.

**Figure 14:**
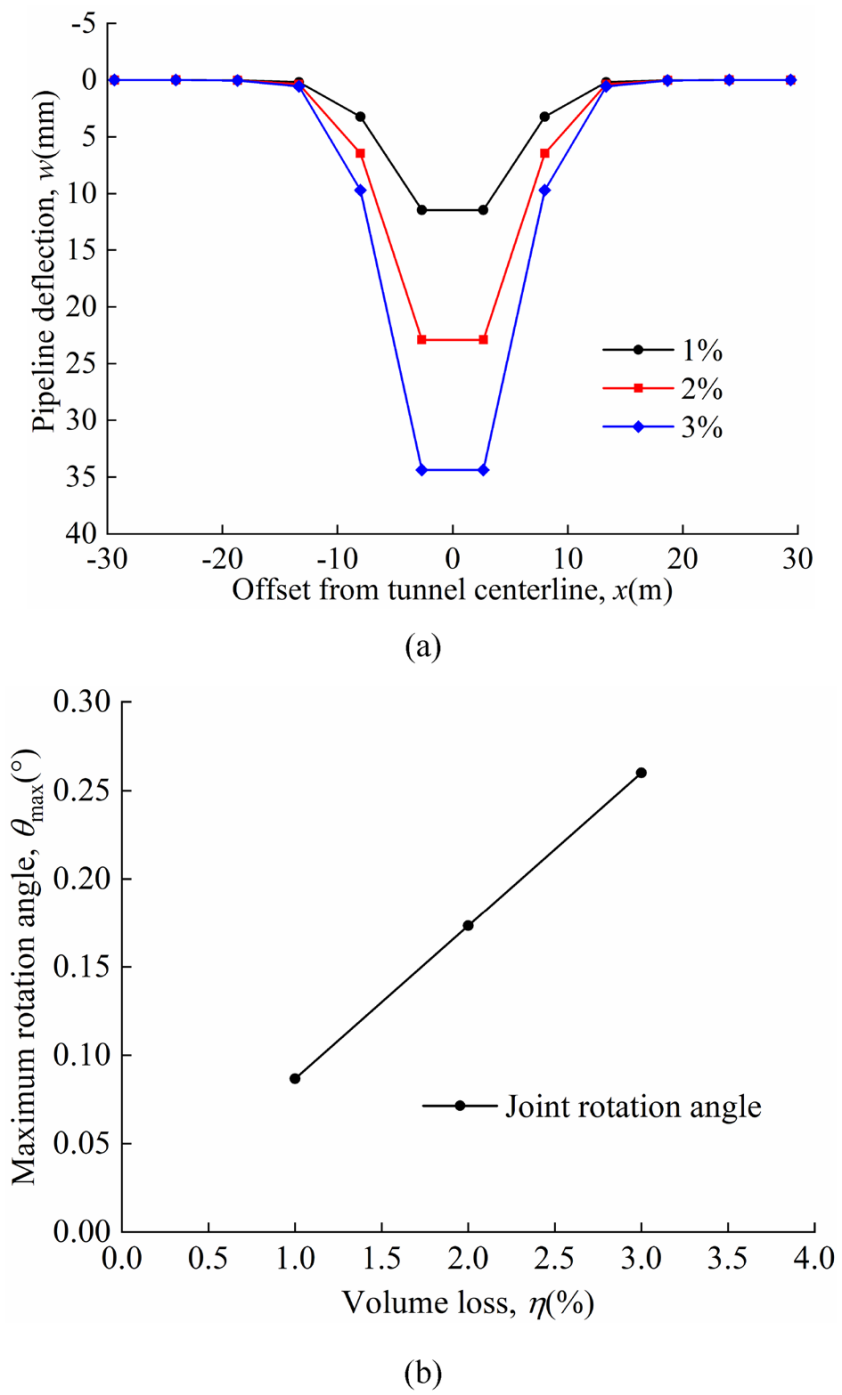
Different volume losses in the "even" case: (**a**) pipeline deflection; (**b**) variation of maximum rotation angle with *η*.

The deflection of the pipeline and the maximum rotation angle of the joint for different volume losses in the "odd" case are shown in Fig. 13a and b; the deflection of the pipeline and the maximum rotation angle of the joint for different volume losses in the "even" case are shown in Fig. 14a and b. The maximum deflection of the pipeline always occurs directly above the tunnel axis and the maximum rotation angle of the joint occurs at the nearest "discontinuous joint" to the tunnel axis. A comparison of Figs. 13a and 14a shows that the maximum pipeline deflection values and maximum rotation angle of the joint in the "odd" case are greater than that in the "even" case for the same calculation parameters. In both the "odd" and "even" cases, the graph shows that as the volume loss increases, the pipeline deflection and the maximum rotation angle of the joint increases linearly. This shows that controlling the volume loss is an effective measure to prevent excessive deformation of the pipeline.

The maximum pipeline deflection and the maximum rotation angle of the joint for different *η* values in the "odd" and "even" cases are shown in Table 4.

**Table 4:** *w*_max_, *θ*_max_, and position of *θ*_max_.

		*η* = 1%	*η* = 2%	*η* = 3%
Odd	*w*_max_(mm)	13.31	26.63	39.94
	θ_max_(°)	0.129	0.257	0.386
	Position of θ_max_(m)	*x* = 0	*x* = 0	*x* = 0
Even	*w*_max_(mm)	11.50	23.00	34.50
	θ_max_(°)	0.087	0.173	0.260
	Position of θ_max_(m)	*x* = ± 0.5*L*	*x* = ± 0.5*L*	*x* = ± 0.5*L*

### Rotational stiffness of "discontinuous joints"

Different rotational stiffnesses of the "discontinuous joint" are analysed (1) *β*_0_ = 4.47 × 10^6^N⋅m/rad; (2) *β*_0_ = 4.47 × 10^8^N⋅m/rad; (3) *β*_0_ = 4.47 × 10^10^N⋅m/rad. The volume loss is *η* = 2%. The other parameters are consistent with those of the model centrifuge tests carried out by Vorster^26^. The pipeline deflection and maximum rotation angle of joints in "odd" and "even" cases have been discussed.

**Figure 15:**
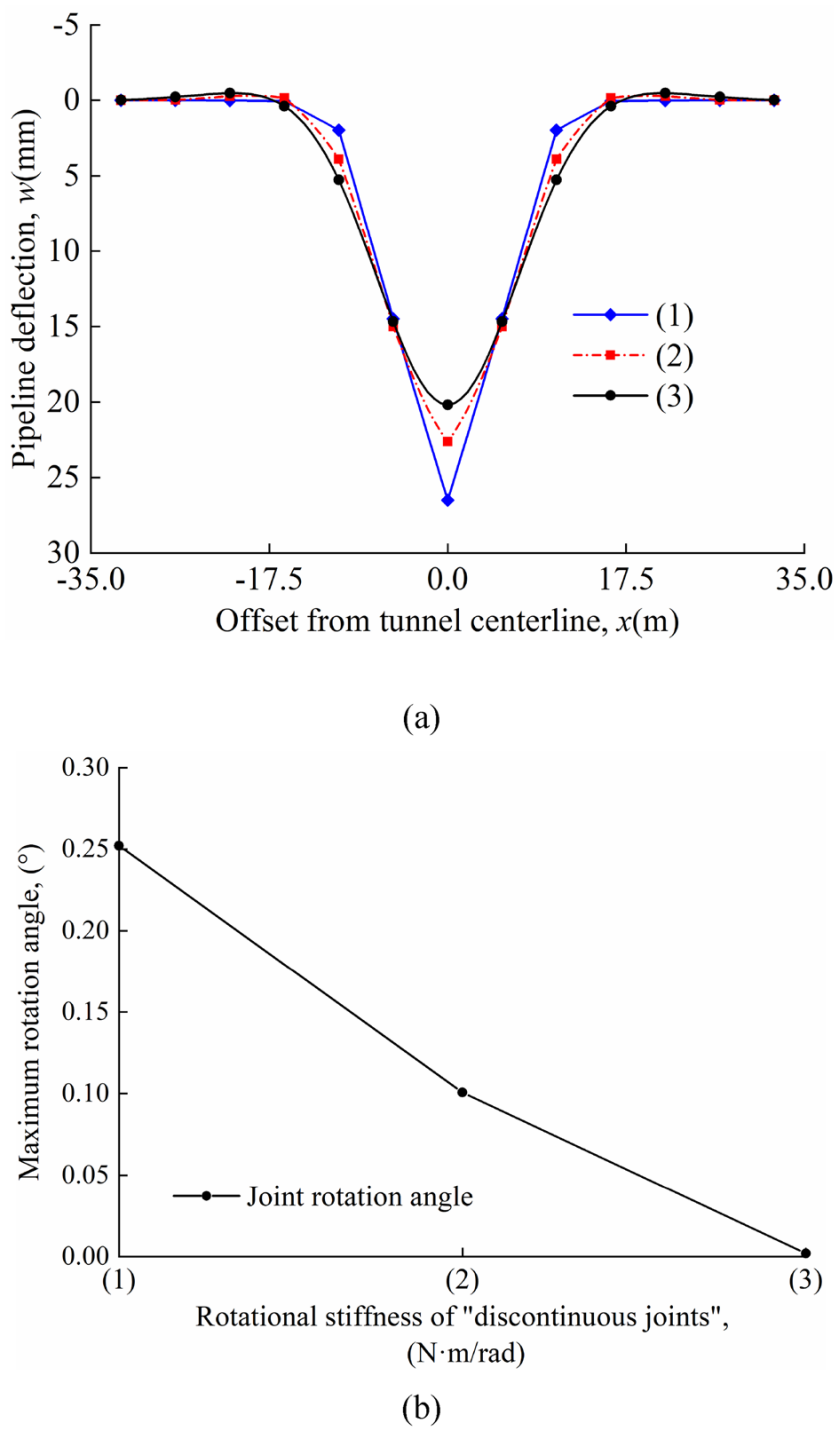
Different rotational stiffnesses in the "odd" case: (**a**) pipeline deflection; (**b**) variation of maximum rotation angle with *β*_0_.

**Figure 16:**
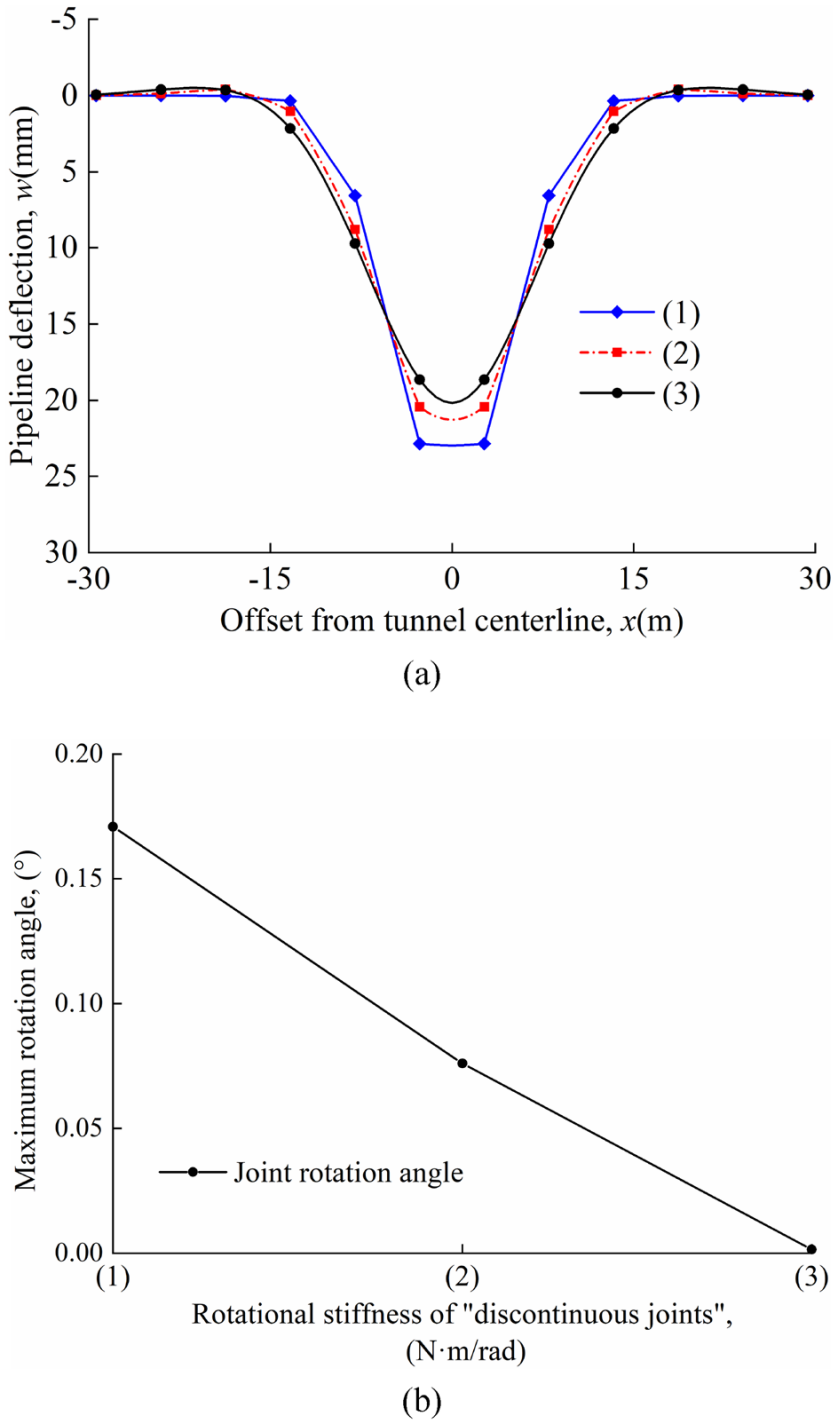
Different rotational stiffnesses in the "even" case: (**a**) pipeline deflection; (**b**) variation of maximum rotation angle with *β*_0_.

The deflection of the pipeline and the maximum rotation angle of the joint for different rotational stiffnesses in the "odd" case are shown in Fig. 15a and b; the deflection of the pipeline and the maximum rotation angle of the joint for different rotational stiffnesses in the "even" case are shown in Fig. 16a and b. In the calculations, it is found that the maximum deflection of the pipeline always occurred directly above the tunnel axis and the maximum rotation angle of the joint occurred at the nearest "discontinuous joint" to the tunnel axis. A comparison of Figs. 15a and 16a shows that the maximum pipeline deflection values and maximum rotation angle of the joint in the "odd" case are greater than that in the "even" case for the same calculation parameters. In the "odd" and "even" cases, the maximum deflection value and the maximum rotation angle of the joint decrease as *β*_0_ increases.

The maximum pipeline deflection and the maximum rotation angle of the joint for different *β*_0_ values in the "odd" and "even" cases are shown in Table 5.

**Table 5:** *w*_max_, *θ*_max_, and position of *θ*_max_.

		*β*_0_ = 4.47 × 10^6^N⋅m/rad	*β*_0_ = 4.47 × 10^8^N⋅m/rad	*β*_0_ = 4.47 × 10^10^N⋅m/rad
Odd	*w*_max_(mm)	26.50	22.62	20.20
	*θ*_max_(°)	0.252	0.100	0.002
	Position of *θ*_max_(m)	*x* = 0	*x* = 0	*x* = 0
Even	*w*_max_(mm)	22.95	21.27	20.16
	*θ*_max_(°)	0.171	0.076	0.001
	Position of *θ*_max_(m)	*x* = ± 0.5*L*	*x* = ± 0.5*L*	*x* = ± 0.5*L*

### Ratio of pipeline section length to inflection point of soil settlement curve

The ratio of pipeline section length to the inflection point of the soil settlement curve are analysed *L*/*i*_s_ = 0.5, 1, 1.5, 2. The volume loss is *η* = 2%. The other parameters are consistent with those of the model centrifuge tests carried out by Vorster^26^. The pipeline deflection and maximum rotation angle of joints in "odd" and "even" cases have been discussed.

**Figure 17:**
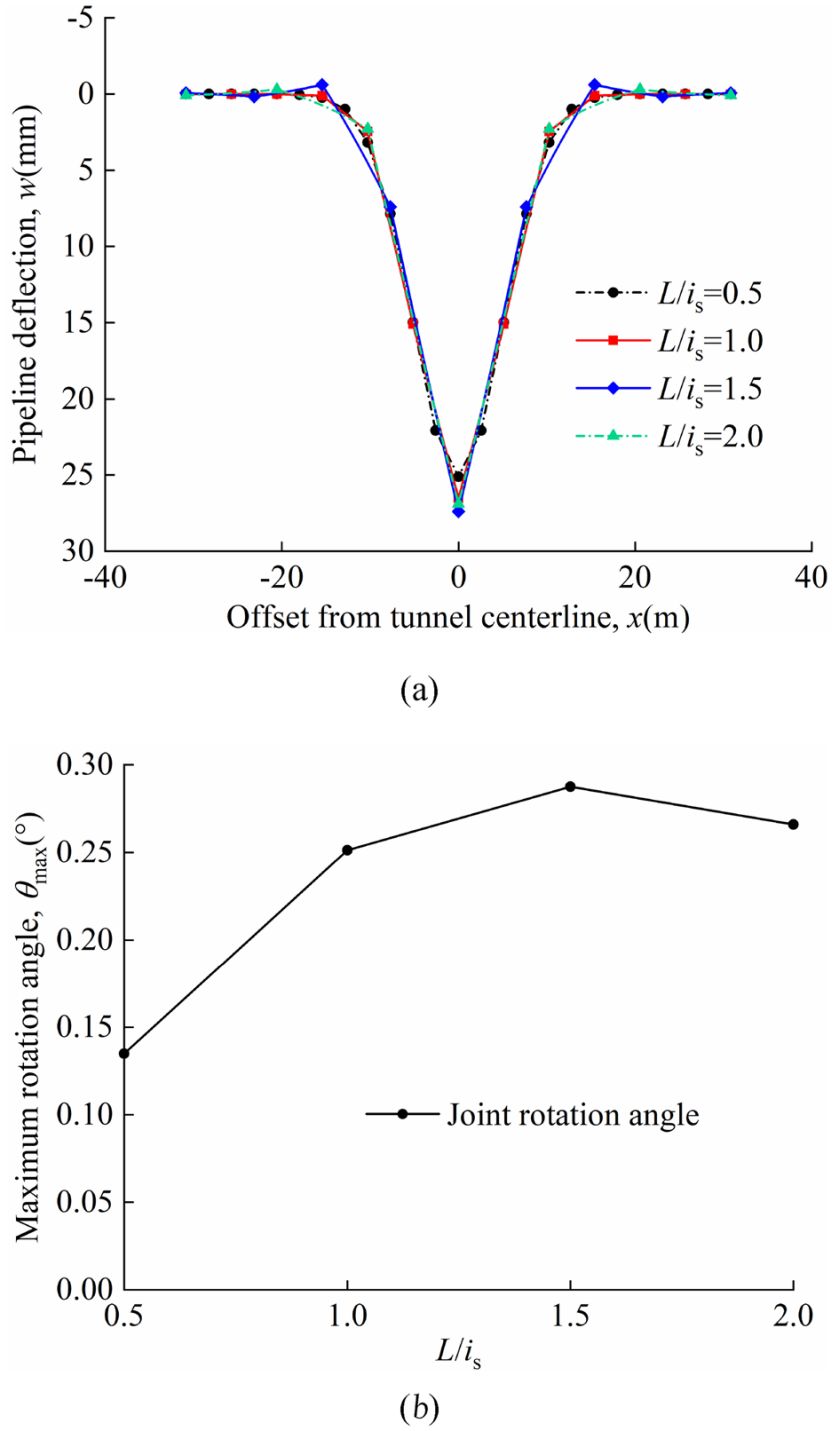
Different ratios of the length of the pipeline section to the inflection point of the soil settlement curve in the "odd" case: (**a**) pipeline deflection; (**b**) variation of maximum rotation angle with *L*/*i*_*s*_.

**Figure 18:**
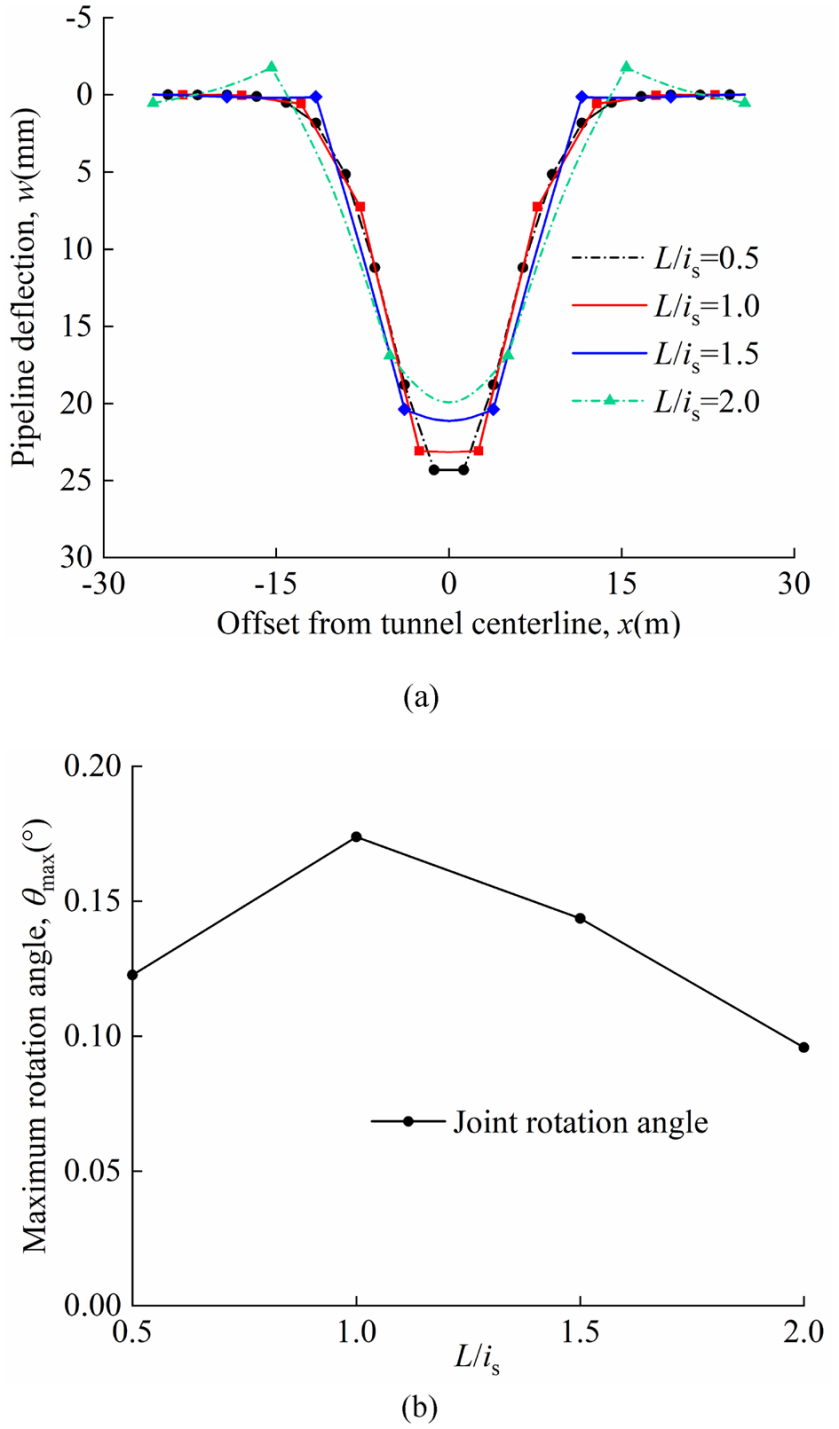
Different ratios of the length of the pipeline section to the inflection point of the soil settlement curve in the "even" case: (**a**) pipeline deflection; (**b**) variation of maximum rotation angle with *L*/*i*_*s*_.

Figure 17a and b show the pipeline deflection and the maximum rotation angle of the joint at different *L*/*i*_s_ for the "odd" case; Fig. 18a and b show the pipeline deflection and the maximum rotation angle of the joint at different *L*/*i*_s_ for the "even" case; In the calculations, it is found that the maximum deflection values of the pipelines always occurs directly above the tunnel axis. In the "odd" case the maximum rotation angle of the joint occurs at the "discontinuous joint" directly above the tunnel axis. In the "even" case, when *L*/*i*_s_ = 0.5, 1, the maximum rotation angle of the joint occurs at *x* =  ± 0.5*L*; when *L*/*i*_s_ = 1.5, 2, the maximum rotation angle of the joint occurs at *x* =  ± 1.5*L*. At *L*/*i*_s_ = 1.5, 2.0, the pipeline bulges and the maximum rotation angle of the joint occurs at the "discontinuous joint" of the bulge. The additional soil loads on the pipeline are mainly concentrated at the tunnel axis location, with less additional soil loads at the shoulder and less soil settlement at the shoulder location. When the pipeline section is longer, there is a situation where the additional load is larger on the side close to the tunnel axis and smaller on the side away from the tunnel axis; this in turn leads to a bulge in the pipeline on the side with the smaller additional load.

A comparison of Figs. 17a and 18a shows that the maximum pipeline deflection values and maximum rotation angle of the joint in the "odd" case are greater than that in the "even" case for the same calculation parameters. In the "odd" case the maximum deflection of the pipeline and the maximum rotation angle of the joint both tend to increase and then decrease as *L*/*i*_s_ increases, with a peak at *L*/*i*_s_ = 1.5. The maximum deflection of the pipeline in the "even" case tends to decrease as the value of *L*/*i*_s_ increases; the maximum rotation angle of the joint tends to increase and then decrease as *L*/*i*_s_ increases, reaching a maximum at *L*/*i*_s_ = 1.

The maximum pipeline deflection and the maximum rotation angle of the joint for different *L*/*i*_*s*_ values in the "odd" and "even" cases are shown in Table 6.

**Table 6:** *w*_max_, *θ*_max_, and position of *θ*_max_.

		*L*/*i*_s_ = 0.5	*L*/*i*_s_ = 1.0	*L*/*i*_s_ = 1.5	*L*/*i*_s_ = 2.0
Odd	*w*_max_(mm)	25.11	26.52	27.40	26.90
	*θ*_max_(°)	0.135	0.251	0.287	0.266
	Position of *θ*_max_(m)	*x* = 0	*x* = 0	*x* = 0	*x* = 0
Even	*w*_max_(mm)	24.32	23.15	21.11	19.92
	*θ*_max_(°)	0.123	0.174	0.144	0.096
	Position of *θ*_max_(m)	*x* = ± 0.5*L*	*x* = ± 0.5*L*	*x* = ± 1.5*L*	*x* = ± 1.5*L*

### Soil elastic modulus

Different soil elastic moduli are analysed *E* = 10 MPa, 30Mpa, 50 MPa. The relative stiffness of pipeline-soil (*E*_p_*I*_p_/(*Ei*_s_^4^)) decreases as *E* increases. The volume loss is *η* = 2%. The other parameters are consistent with those of the model centrifuge tests carried out by Vorster^26^. The pipeline deflection and maximum rotation angle of joints in "odd" and "even" cases have been discussed.

**Figure 19:**
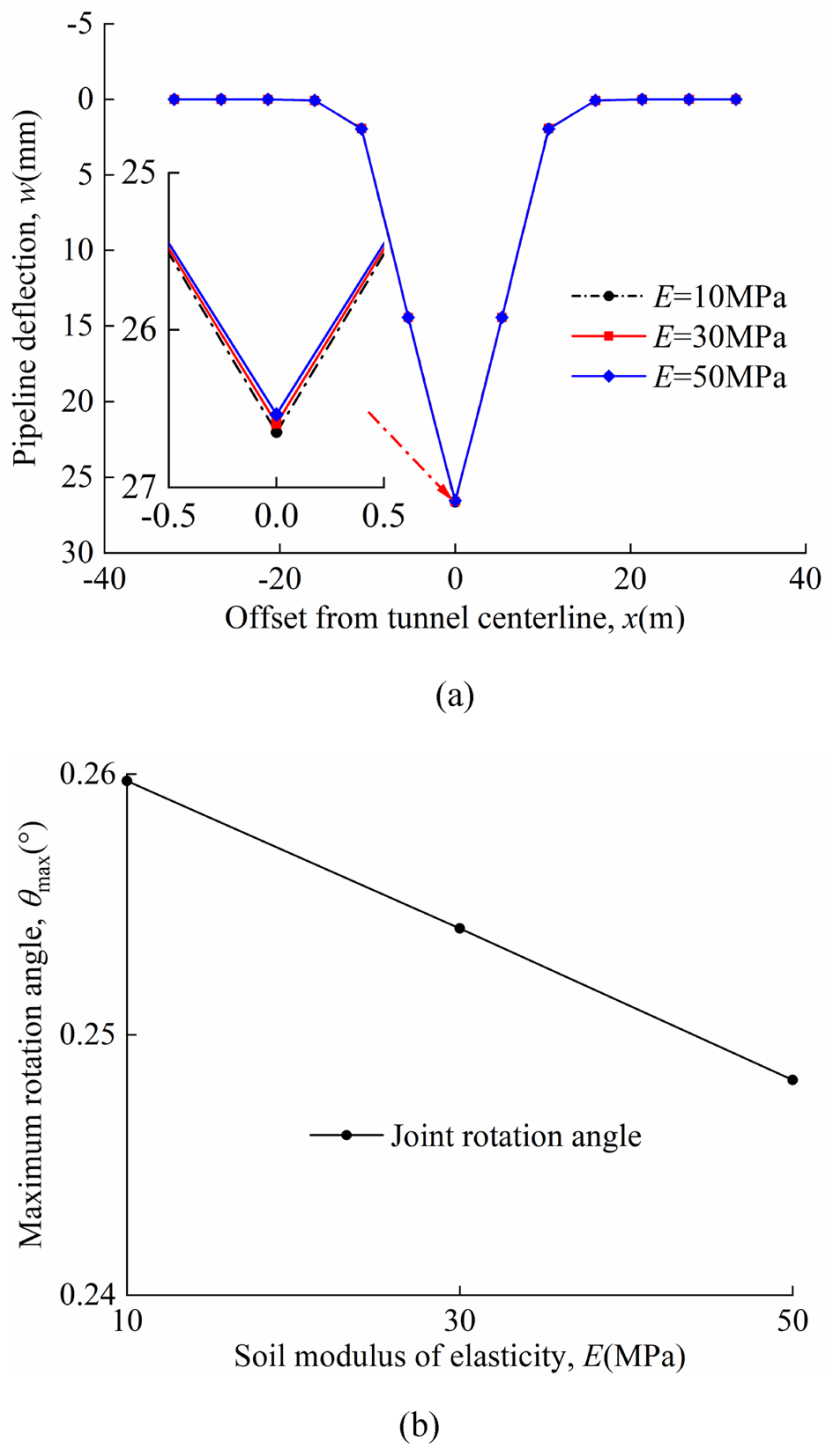
Different soil elastic moduli in the "odd" case: (**a**) pipeline deflection; (**b**) variation of maximum rotation angle with *E*.

**Figure 20:**
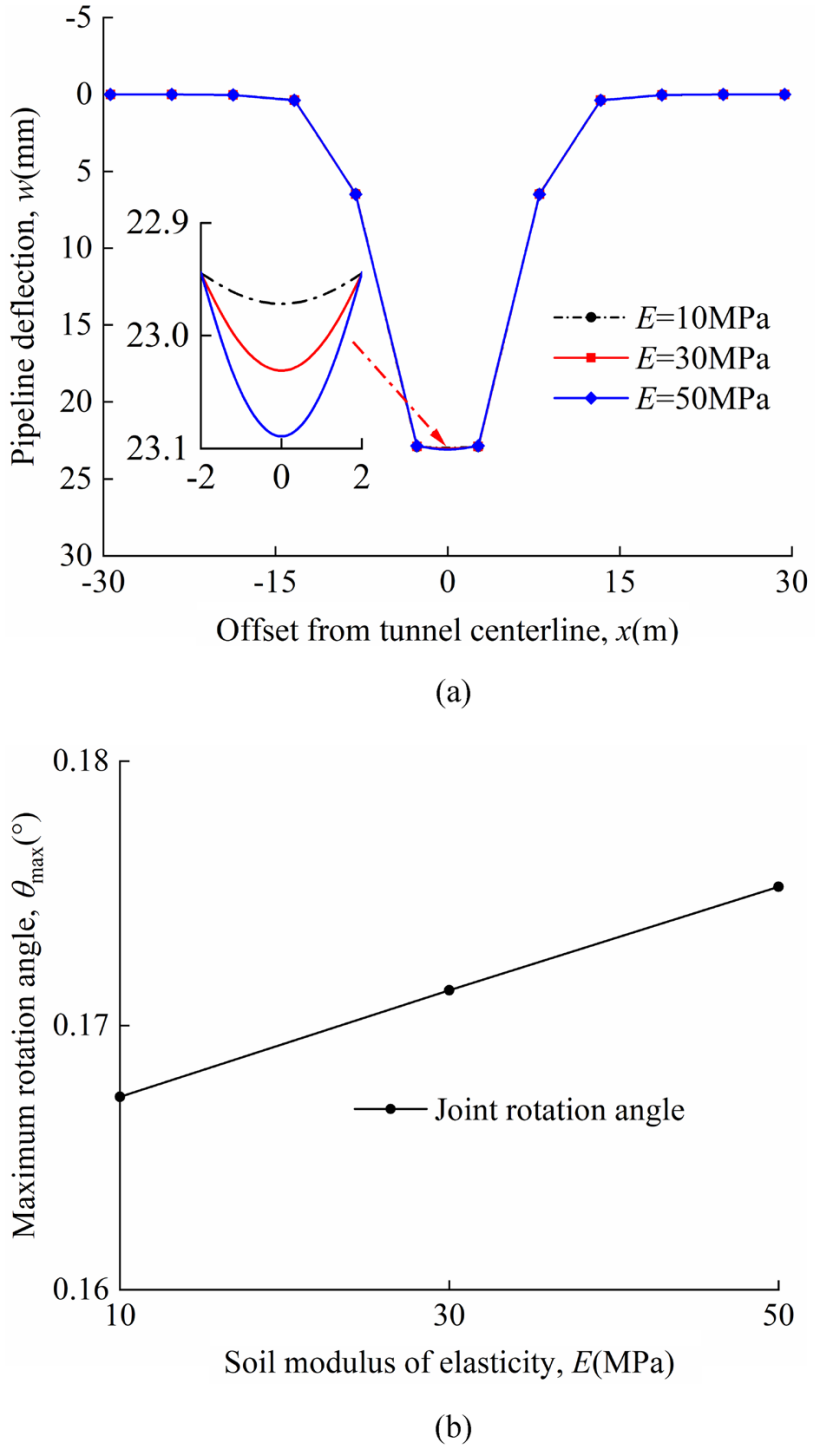
Different soil elastic moduli in the "odd" case: (**a**) pipeline deflection; (**b**) variation of maximum rotation angle with *E*.

The deflection of the pipeline and the maximum rotation angle of the joints for different soil elastic moduli *E* in the "odd" case are shown in Fig. 19a and b; the deflection of the pipeline and the maximum rotation angle of the joints for different soil elastic moduli *E* in the "even" case are shown in Fig. 20a and b. In the calculations, it is found that the maximum deflection of the pipeline always occurs directly above the tunnel axis and the maximum rotation angle of the joint occurs at the nearest "discontinuous joint" to the tunnel axis. A comparison of Figs. 19a and 20a shows that the maximum pipeline deflection values and maximum rotation angle of the joint in the "odd" case are greater than that in the "even" case for the same calculation parameters. In the "odd" case, the maximum deflection value of the pipeline and the maximum rotation angle of the joint decrease as *E* increases. In the "even" case, the maximum deflection value of the pipeline and the maximum rotation angle of the joint increase as *E* increases. It can be seen from the graph that the change in *E* has little effect on the deformation of the discontinuous pipeline.

The maximum pipeline deflection and the maximum rotation angle of the joint for different *E* values in the "odd" and "even" cases are shown in Table 7.

**Table 7:** *w*_max_, *θ*_max_, and position of *θ*_max_.

		*E* = 10 MPa	*E* = 30 MPa	*E* = 50 MPa
Odd	*w*_max_(mm)	26.65	26.60	26.54
	*θ*_max_(°)	0.260	0.254	0.248
	Position of *θ*_max_(m)	*x* = 0	*x* = 0	*x* = 0
Even	*w*_max_(mm)	22.97	23.03	23.09
	*θ*_max_(°)	0.167	0.171	0.175
	Position of *θ*_max_(m)	*x* = ± 0.5*L*	*x* = ± 0.5*L*	*x* = ± 0.5*L*

## Conclusions

Most of literatures have done on continuous pipelines but less on discontinuous pipelines, and for this reason that the deformation of discontinuous pipeline caused by tunnel excavation is studied in this paper. The greenfield settlement of the soil at the buried position of the pipeline is used as an additional load applied to the existing pipeline. Based on the rigid bar method, the pipeline is divided into equal-length pipeline units, and the support of the soil on the pipeline is regarded as a spring support concentrated at the joint position, using a simply supported beam as the basic system. The knowledge of structural mechanics is used to solve for vertical displacement and rotation angle of the joint, whereby a new method that can calculate the deformation of continuous and discontinuous pipelines is proposed. The effectiveness of the method in this paper is verified by comparison with engineering examples and centrifuge test results, and the influencing factors of pipeline deformation are analyzed. The main conclusions are as follows:

1. The additional load acting on the pipe increases as the volume loss increases, so the maximum pipeline deflection and the maximum rotation angle of the joint both increase linearly. The stiffness of the discontinuous pipeline to resist deformation increases as the rotational stiffness of the "discontinuous joint" increases, so that the maximum deflection of the pipe and the maximum angle of rotation of the joint both decrease.
2. In the "odd" case, when the pipeline section length is short (*L*/*i*_s_ < 1.5), the ability of the pipeline to resist deformation is weak, and the deformation of the pipeline is close to the deformation of the soil; at this stage, the difference between the pipeline deformation and the soil deformation increases with the increase of the pipeline section length, and the maximum pipeline deflection and the maximum rotation angle of the joint gradually increase. The pipeline section has a certain ability to resist deformation, when the pipeline section length is long (*L*/*i*_s_ > 1.5), the integrity of the pipeline is enhanced with the increase of the pipeline section length, and the maximum pipeline deflection and the maximum rotation angle of the joint gradually decrease. In the "even" case, the integrity of the pipeline increases with the increase of the pipeline section length, the maximum pipeline deflection gradually decreases, the pipeline section has a certain ability to resist deformation, and the maximum rotation angle of the joint first increases and then decreases.
3. As *E* increases, the relative stiffness of pipeline-soil (*E*_p_*I*_p_/(*Ei*_s_^4^)) decreases gradually and the coordination between pipeline and soil increases, so the pipeline deformation gradually approaches that of the soil. Therefore, the maximum pipeline deflection and the maximum rotation angle of the joint gradually decrease in the "odd" case, while the maximum pipeline deflection and the maximum rotation angle of the joint gradually increase in the "even" case.
4. For the same calculated parameters, the maximum pipeline deflection and the maximum rotation angle of the joint in the "odd" case are larger than that in the "even" case. The reason for this is that the rotational stiffness of the "continuous joint" is greater than that of the "discontinuous joint".

## Limitations

This study assumes that the pipeline is not affected by lateral earth pressures and only considers its influence by soil displacement loads, whereas in actual engineering the pipeline is also affected by tunnel excavation and soil structure. The authors assume that the soil is homogeneous, but in reality the soil is mostly non-homogeneous and the foundation soil is anisotropic, stratified and elastic–plastic. Furthermore, the formation displacement has three-dimensional characteristics, which should include both vertical and horizontal displacement, but only the vertical displacement is considered in this study. This study treats the tunnel-pipeline-soil interaction as a plane problem and does not consider the 3D interaction between the tunnel and the underground structure.

## Data Availability

All data, models, or code that support the findings of this study are available from the corresponding author upon reasonable request.

## List of symbols

*x*: Horizontal distance to tunnel axis
*S*(*x*), *S*_max_: Soil settlement at position *x* and the maximum value of soil settlement, respectively
*R*: Tunnel radius
*η*: Volume loss
*i*_s_: Distance between the inflection point of the soil settlement curve and the centre of symmetry of the settlement curve
*z*_s_: Distance from soil layer to surface
*H*: Tunnel depth
*K*: Modified soil modulus of elasticity
*l*: Length of pipeline unit
*β*, *β*_0_: Rotational stiffness of "continuous joints" and "discontinuous joints", respectively
*L*: Pipeline section length
λ: Number of pipeline sections divided
*M*_i,m_: Bending moment of the (i, m)th joint
θ_i,m_, θ_max_: Rotation angle of the (i, m)th joint, maximum angle of rotation of the joint
*p*_i,m_: Spring support reaction force at the (i, m)th joint
*w*_i,m_: Vertical displacement of the (i, m)th joint
*E*_p_*I*_p_: Pipeline bending stiffness
*E*: Soil elastic modulus
*v*: Soil poisson's ratio
*d*: Pipeline diameter
*z*_p_: Burial depth of pipeline
*p*_s_^l^, *p*_s_^r^: Reaction forces provided to the joint by the simply supported beams on either side of the joint
θ_s_: Angle of rotation of the joint s
*S*_l_, *S*_s_, *S*_r_: Vertical settlements of the soil at the joints
*M*_l_, *M*_s_, *M*_r_: Bending moments of joints *l*, *s*, *r*
*β*_s_: Rotational stiffness of the joint s
